# STING aggravates ferroptosis-dependent myocardial ischemia-reperfusion injury by targeting GPX4 for autophagic degradation

**DOI:** 10.1038/s41392-025-02216-9

**Published:** 2025-04-25

**Authors:** Xiaohong Wang, Tao Chen, Sizhe Chen, Jie Zhang, Liangyu Cai, Changhao Liu, Yujie Zhang, Xiao Wu, Na Li, Zhiyong Ma, Lei Cao, Qian Li, Chenghu Guo, Qiming Deng, Wenqian Qi, Yonghao Hou, Ruiqing Ren, Wenhai Sui, Haonan Zheng, Yun Zhang, Meng Zhang, Cheng Zhang

**Affiliations:** State Key Laboratory for Innovation and Transformation of Luobing Theory; Key Laboratory of Cardiovascular Remodeling and Function Research of MOE, NHC, CAMS and Shandong Province; Department of Cardiology, Qilu Hospital of Shandong University, Jinan, 250012 China

**Keywords:** Cardiology, Cardiovascular diseases

## Abstract

Despite advancements in interventional coronary reperfusion technologies following myocardial infarction, a notable portion of patients continue to experience elevated mortality rates as a result of myocardial ischemia-reperfusion (MI/R) injury. An in-depth understanding of the mechanisms underlying MI/R injury is crucial for devising strategies to minimize myocardial damage and enhance patient survival. Here, it is discovered that during MI/R, double-stranded DNA (dsDNA)-cyclic GMP-AMP synthase (cGAS)-stimulator of interferon genes (STING) signal accumulates, accompanied by high rates of myocardial ferroptosis. The specific deletion of *cgas* or *Sting* in cardiomyocytes, resulting in the inhibition of oxidative stress, has been shown to mitigate ferroptosis and I/R injury. Conversely, activation of STING exacerbates ferroptosis and I/R injury. Mechanistically, STING directly targets glutathione peroxidase 4 (GPX4) to facilitate its degradation through autophagy, by promoting the fusion of autophagosomes and lysosomes. This STING-GPX4 axis contributes to cardiomyocyte ferroptosis and forms a positive feedback circuit. Blocking the STING-GPX4 interaction through mutations in *T*267 of STING or *N*146 of GPX4 stabilizes GPX4. Therapeutically, AAV-mediated GPX4 administration alleviates ferroptosis induced by STING, resulting in enhanced cardiac functional recovery from MI/R injury. Additionally, the inhibition of STING by H-151 stabilizes GPX4 to reverse GPX4-induced ferroptosis and alleviate MI/R injury. Collectively, a novel autophagy-dependent ferroptosis mechanism is identified in this study. Specifically, STING autophagy induced by anoxia or ischemia-reperfusion leads to GPX4 degradation, thereby presenting a promising therapeutic target for heart diseases associated with I/R.

## Introduction

Ischemic heart disease remains the predominant cause of morbidity and mortality globally. Despite timely interventional coronary reperfusion being effective in salvaging ischemic myocardium, myocardial ischemia-reperfusion (MI/R) injury remains a significant contributor to mortality in a substantial patient population.^1^ To date, despite intense decades-long research, no specific MI/R injury targeting agent has arrived at the clinical area.^2^ There exists an urgent medical imperative to comprehensively and deeply understand the intricate mechanisms that govern cardiomyocyte death. Grasping these mechanisms is fundamental as it can unlock the discovery of novel and innovative therapeutic targets, which are of utmost importance for the efficient and effective management of the associated pathological state.

Cell death, especially the death of cardiomyocytes (CMs), is a crucial aspect within the pathophysiology of cardiovascular diseases. Among the forms of cell death, ferroptosis, an iron-dependent cell death modality marked by excessive lipid peroxidation, has emerged as a key factor in MI/R injury pathophysiology.^3^ Ferroptosis is regulated by the metabolism of iron, lipids, amino acids, and glutathione, and is closely associated with multiple heart conditions. This makes targeting ferroptosis a promising therapeutic approach for MI/R injury, and thus potentially revolutionizing treatment strategies for this common and life-threatening condition. Glutathione peroxidase 4 (GPX4), having the function of converting lipid peroxides into lipid alcohols with less harmfulness, has become a pivotal regulatory factor in inhibiting ferroptosis. Previous studies have demonstrated that ferroptosis triggered by MI/R is concomitant with the suppression of GPX4. During the MI/R process, a reduction in GPX4 levels coincides with the initiation of ferroptosis. In contrast, elevating GPX4 levels effectively mitigates myocardial injury and enhances cardiac function.^4,5^ However, the precise regulation of GPX4 protein levels and its underlying degradation mechanism remain elusive. Therefore, the discovery of factors that regulate GPX4 might present an attractive therapeutic target for the modulation of ferroptosis occurring during the MI/R process.

The cyclic guanosine monophosphate (GMP)-adenosine monophosphate (AMP) (cGAMP) synthase (cGAS) and stimulator of interferon genes (STING) stand as pivotal and highly conserved immune components within the mammalian immune system. They are integral in the body’s defense mechanism against pathological nucleic acids, with a particular focus on double-stranded DNA (dsDNA).^6^ Upon abnormal DNA binding, cGAS produces a second messenger cGAMP. This newly synthesized cGAMP then engages in a specific interaction with STING, promoting its conformational changes. Once activated, STING binds to TANK-binding kinase 1 (TBK1) and interferon regulatory factor 3 (IRF3). This binding occurrence sets off a complex cascade of molecular events, which ultimately culminate in the initiation of interferon (IFN) expression.^7–9^ The production of IFN not only contributes to the body’s inflammatory response but also elicits powerful antiviral effects. In recent years, an increasing number of studies have reported that STING-associated signaling dysregulation can be observed in a wide array of human pathologies. These include, but are not limited to, type I IFN diseases,^10^ where the normal IFN signaling pathways are disrupted, and in cancer,^11^ where STING regulates anticancer immunity in both IFN-dependent and IFN-independent manners. Moreover, many reports revealed that STING functions independently of classical antiviral pathways, such as in aging^12^ and autophagy.^13^ STING plays a role in driving tissue senescence and premature aging-related tissue degeneration, and autophagy represents an ancestral function intrinsic to the cGAS-STING pathway. Indeed, a large part of disease progression and cell death modulated by STING occurs independently of IRF3 and STING’s antiviral gene expression. The development of STING-associated vasculopathy occurs without relying on IRF3.^14^ Human STING, which functions as a proton channel, has its interferon-inducing ability separated from its key roles in promoting LC3B lipidation and inflammasome activation.^15^ In phagosomes, STING directly interacted with Src, and this interaction hindered Src from recruiting Syk and phosphorylating it.^16^ The exploration of STING’s non-traditional immune functions is poised to substantially enrich our comprehension of immune complexity.

In our investigation, we explored how STING modulates myocardial ferroptosis when the heart undergoes MI/R injury. I/R-induced mitochondrial damage leads to the release of dsDNA, which is subsequently recognized by cGAS, resulting in the production of the second messenger cGAMP and activation of STING. STING can directly trigger myocardial ferroptosis via its interaction with GPX4. Once STING binds to GPX4, it prompts augmented autophagy, which in turn causes the subsequent degradation of GPX4, fueling the process of myocardial ferroptosis. We also evaluated two effective therapeutic strategies against MI/R injury, including the utilization of AAV-mediated GPX4 overexpression and the administration of STING antagonists. In the light of our research, the results we’ve obtained offer fresh perspectives on the development mechanism of ferroptosis and establish STING as a potential therapeutic target to prevent MI/R injury, to improve patient outcomes and to reduce mortality rates associated with ischemic heart disease.

## Results

### I/R triggers cGAS-STING upregulation in CMs

**Fig. 1:**
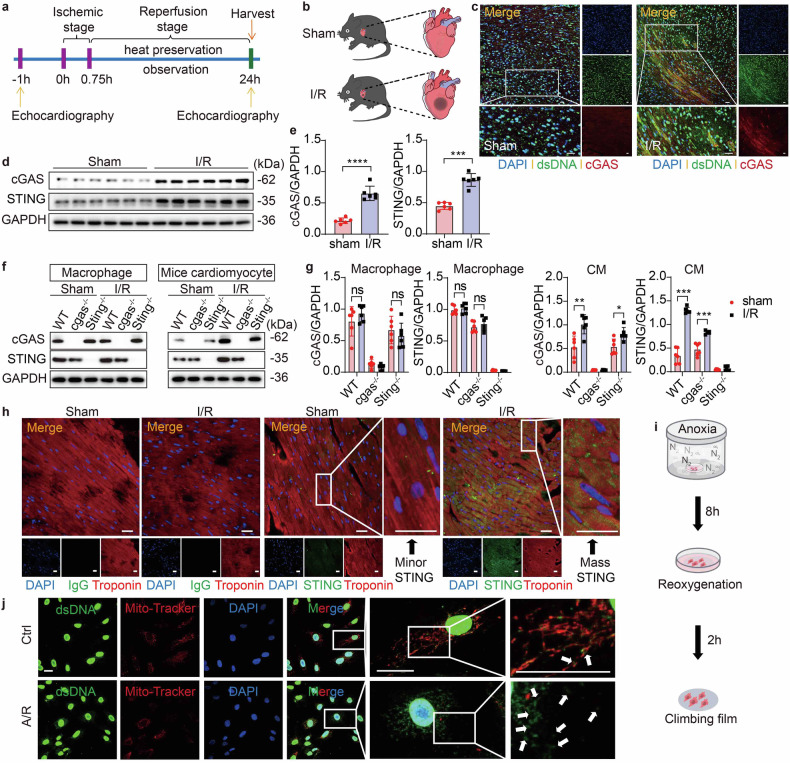
I/R triggers cGAS-STING upregulation in CMs. Male mice (aged 8 weeks) were subjected to the operation and euthanized at 24 h post-I/R or Sham. **a** Display the mouse I/R model operation flowchart. **b** Schematic diagram showing the mouse I/R injury model. **c** Double immunofluorescence analysis for detecting dsDNA and cGAS in the same heart section of the WT border region post I/R or Sham. The positive reactions of tissue sections are displayed in green (dsDNA) and red (cGAS). The positive reaction for co-localisation is displayed in yellow. Scale bar = 20 μm. **d**, **e** Western blot and quantification of cGAS and STING in WT cells isolated from border region post I/R or Sham (*n* = 6). **f**, **g** Western blot and quantification of cGAS and STING in various cell types isolated from the heart border region of *cgas*^−/−^, *Sting*^*−/−*^, or WT mice post I/R or Sham (*n* = 6). **h** Double immunofluorescence analysis for detecting markers of CMs and STING in the same heart section of the WT border region post I/R or Sham. The positive reactions of tissue sections are displayed in green (STING) and red (Troponin). The positive reaction for co-localisation is displayed in yellow. Scale bar = 20 μm. **i** Schematic diagram showing the CM A/R operation flowchart. **j** Double immunofluorescence analysis for detecting dsDNA and mitochondria (labeled with Mito-Tracker) in MPCs post-A/R. The positive reactions are displayed in green (dsDNA) and red (Mito-Tracker). The positive reaction for co-localisation is displayed in yellow. Arrowheads indicate released dsDNA. Scale bar = 10 μm. Data are expressed as the mean ± SEM. NS non-significant, ***P < 0.001 and ****P < 0.0001 (unpaired two-tailed Student’s *t* test). I/R Ischemia reperfusion, dsDNA double-stranded DNA, A/R anoxia/ reoxygenation, cGAS cyclic guanosine monophosphate-adenosine monophosphate synthase, STING stimulator of interferon genes, WT wild type, *cgas*^−/−^ *cgas* knockout mice, *Sting*^−/−^, *Sting* knockout mice, CM cardiomyocyte

To begin to define effects of cGAS-mediated DNA sensing in I/R injury, we first tested for changes in cytosolic DNA levels. To elucidate whether heart I/R caused cell DNA damage, we initially set up an I/R model using male *C57BL/6J* mice that were 8 weeks old (Fig. 1a, b) and analyzed the coimmunostaining and expression of double-stranded DNA (dsDNA) and cGAS in the I/R border region. Markedly increased amounts of cytosolic DNA and cGAS activation were observed in cells from mice undergoing I/R compared to Sham-operated controls (Fig. 1c), suggesting profound DNA damage and the leakage of DNA into the cytosol in diseased tissues. Following the recognition of dsDNA, cGAS undergoes activation. To further investigate, we employed Western blot assays to assess the abundance of cGAS and STING proteins. The results revealed a significant upregulation of both cGAS and STING expression in the I/R border region, occurring after 45 min of heart ischemia followed by 24 h of reperfusion (Fig. 1d, e). However, cGAS and STING protein levels in the infarct core did not show significant changes compared to the Sham group (Supplementary Fig. 1a), and the variations were negligible in the non-ischemic region, which remains unaffected by I/R (Supplementary Fig. 1b). In parallel, the immunofluorescence staining results for tissue sections from both the I/R and Sham groups indicated that the expression of STING was markedly enhanced within the ischemic region (border region) of the I/R group but showed no significant changes in the infarct core or non-ischemic region (supplementary Fig. 1c).

In addition, to detect evidence of cGAS-STING pathway activation post-I/R, we isolated CMs, fibroblasts and macrophages populations from the hearts of WT, *cgas*^*−/−*^ or *Sting*^*−/−*^ mice following established protocols, which allowed us to investigate the specific roles of cGAS and STING in different cardiac cell types post-I/R. The upregulation of cGAS and STING in CMs from the border region of the I/R heart was confirmed at the protein level. Notably, these increases were negligible in *cgas*- or *Sting*-null CMs (Fig. 1f, g). However, no apparent differences were observed in the macrophages or fibroblasts, regardless of whether they underwent I/R (Fig. 1f, g, Supplementary Fig. 1d). Since the results mentioned above implied a dependency of cGAS’s role in I/R on STING, we next shifted our focus to STING. Then we devised an immunofluorescence co-localization experiment to further elucidate the expression pattern of STING in CMs. The immunofluorescence staining revealed a significant upregulation of STING expression in CMs located in the border region of the I/R heart (Fig. 1h), in contrast to the negative control.

To verify our results ex vivo, we established an anoxia/reoxygenation (A/R) model with mouse primary CMs (MPCs) obtained from mouse hearts (Fig. 1i). The coimmunostaining analysis of live-cell mitochondrial probes (Mito-Tracker) and dsDNA was used to determine the cytosolic DNA that did not colocalize with either the nuclei or mitochondria. Remarkably, we observed a significant release of dsDNA, accompanied by severe mitochondrial damage manifesting as enlargement, shortening, and thickening post A/R (Fig. 1j), in contrast to the control MPCs.

Together, these findings point out that dsDNA-cGAS-STING signaling is activated in the heart CMs during I/R. We directed our focus towards understanding the specific role of cGAS and STING in CMs during I/R injury.

### Deletion of cGAS-STING protects against MI/R injury

**Fig. 2:**
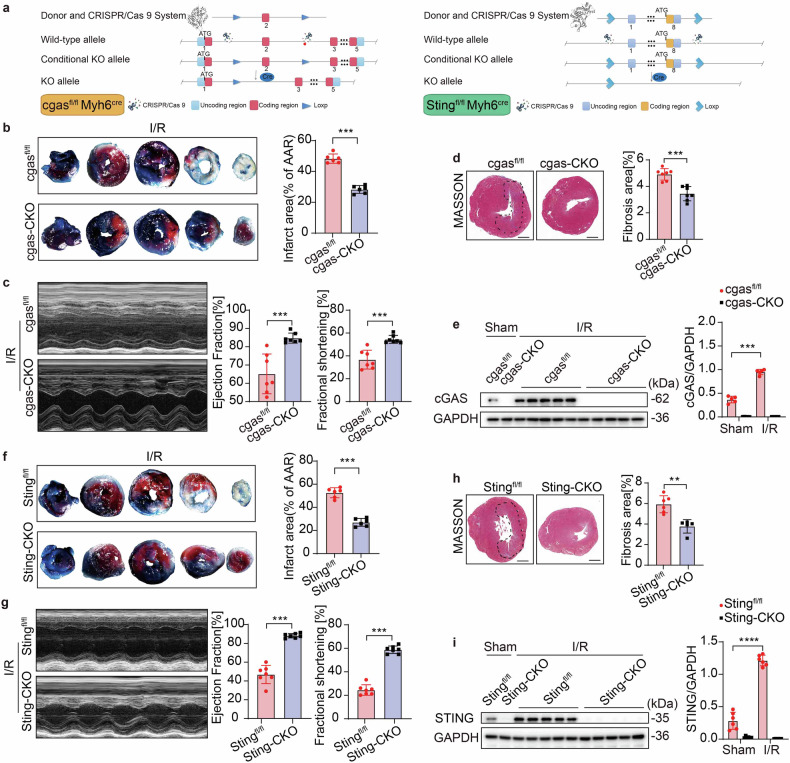
Deletion of cGAS-STING protects against MI/R injury. **a** Structural pattern diagram of *cgas* or *Sting* CM-specific (*Myh6*-iCre) conditional knockout mice. **b**–**e** Effect of *cgas*-CKO on myocardial infarct size, FS, EF and fibrosis area following I/R or Sham: **b** Myocardial infarct size (% of AAR) with representative tissue sectioning (*n* = 6); **c** Echocardiography and measured EF%, and FS% (*n* = 7); **d** Masson staining and measured fibrosis area% (*n* = 7), scale bar=1 mm; **e** Western blot and quantification of cGAS in CMs isolated from the heart border region (*n* = 5). **f**–**i** Effect of *Sting*-CKO on myocardial infarct size, FS, EF and fibrosis area following I/R: **f** Myocardial infarct size (% of AAR) with representative tissue sectioning (*n* = 6); **g** Echocardiography and measured EF%, and FS% (*n* = 7); **h** Masson staining and measured fibrosis area% (*n* = 6), scale bar = 1 mm; **i** Western blot and quantification of STING in CMs isolated from the heart border region (*n* = 6). Mean ± SEM, **P < 0.01, ***P < 0.001, and ****P < 0.0001. AAR area at risk, IF infarct area, *cgas*-CKO *cgas*^fl/fl^ *Myh*^6iCre^, *Sting*-CKO *Sting*^fl/fl^ *Myh*^6iCre^, FS fraction shortening, EF ejection fraction, KO knockout

The role of cGAS or STING in CMs is poorly understood. Next, we generated CM-specific *cgas*-knockout [*cgas*^fl/fl^ *Myh6*^cre^ (*cgas*-CKO)] and *Sting*-knockout [*Sting*^fl/fl^ *Myh6*^cre^ (*Sting*-CKO)] mice for the investigation of the role and functionality of CM-specific cGAS and STING in MI/R injury (Fig. 2a). *cgas*^fl/fl^ and *cgas*^fl/fl^ *Myh6*^cre^, as well as *Sting*^fl/fl^ and *Sting*^fl/fl^ *Myh6*^cre^ were subjected to I/R. Heart injury was assessed by quantifying the area of necrosis in heart sections, assessing cardiac function, and measuring Masson staining. Cardiac function at baseline, represented by left ventricular ejection fraction and fractional shortening (LVEF and FS respectively), was comparable between the KO and their control animals, with no significant differences observed (Supplementary Fig. 1e–h). These findings confirm that the knockout animals do not exhibit intrinsic abnormalities in cardiac function prior to injury. According to the results, *cgas*-CKO mice exhibited a reduced necrosis area, much better cardiac function (EF and FS levels) and decreased fibrosis area following I/R compared to their control mice (Fig. 2b–d), indicating a protective role of CM-specific *cgas* knockout against MI/R injury. Masson staining of 7-day-reperfusion heart tissue showed reduced fibrosis in *cgas*- or *Sting*-CKO mice (Supplementary Fig. 1i, j).

To further delineate the role of cGAS in MI/R injury, we isolated CMs from *cgas*-CKO mice and their control mice of the in vivo I/R model. Western blot analysis revealed that the expression level of cGAS in CMs was minimal under physiological conditions, whereas it became inducible following I/R (Fig. 2e). Importantly, this cGAS upregulation in CMs triggered by I/R was absent in *cgas*-CKO CMs.

Interestingly, similar findings were also observed in *Sting*-CKO mice (Fig. 2f–h). Since STING is a downstream effector of cGAS, the absence of *Sting* in *Sting*-CKO mice led to reduced necrosis area, better cardiac function, and smaller fibrosis area compared to their control mice. I/R could also induce STING expression in *Sting*^fl/fl^ CMs (Fig. 2i). Notably, I/R had no effect on STING protein level in *Sting*-CKO CMs. These findings indicated that cGAS-STING plays a destructive role in cardiac injury post-I/R, and the exacerbation of MI/R injury by cGAS is dependent on the activation of STING.

### STING amplifies myocardial ferroptosis through modulation of oxidative stress injury

**Fig. 3:**
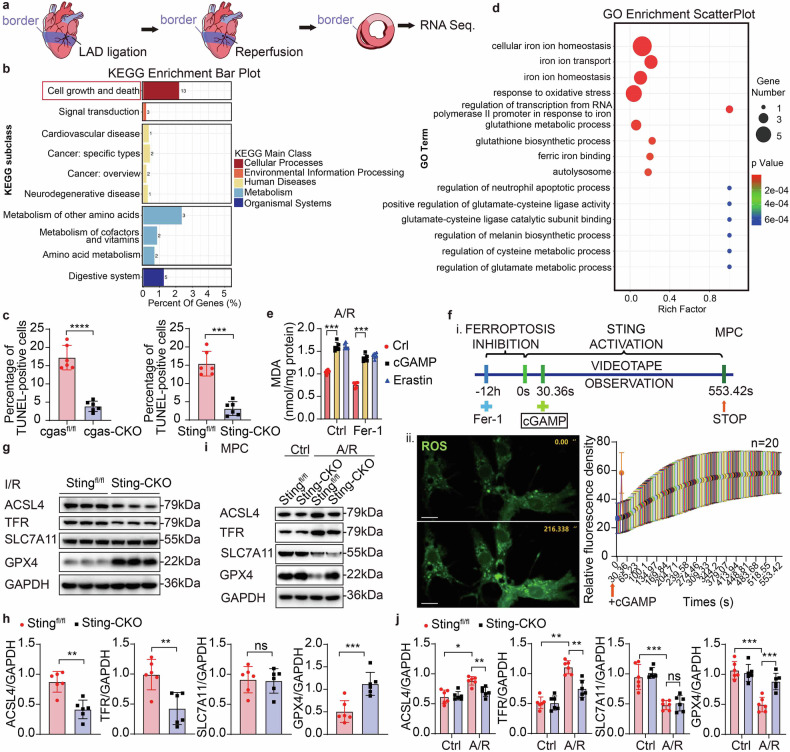
STING amplifies myocardial ferroptosis through modulation of oxidative stress injury. **a** Heart region for RNA-seq post I/R. **b** KEGG Enrichment Bar Plot of RNA-seq. **c** Statistical chart of immunofluorescence image for detecting Tunel in the heart section of the border region of *cgas*-CKO or *Sting*-CKO mice and their control mice post-I/R (*n* = 6). The corresponding image is supplementary Fig. 2a, b. **d** GO Enrichment Scatter Plot of RNA-seq. **e** MDA analysis for detecting lipid peroxidation (*n* = 6). **f** Live cell immunofluorescence imaging analysis for detecting ROS in MPC. Scale bar = 10 μm. **g**, **h** Western blot and quantification of ACSL4, TFR, SLC7A11 and GPX4 in *Sting*^fl/fl^ or *Sting*-CKO CMs post-I/R (*n* = 6). **i**, **j** Western blot and quantification of ACSL4, TFR, SLC7A11 and GPX4 in *Sting*^fl/fl^ or *Sting*-CKO MPCs post-A/R or normoxia (*n* = 6). Mean ± SEM, *P < 0.05, ***P < 0.001, and ****P < 0.0001. ROS reactive oxygen species, Fer-1 ferrostatin-1, cGAMP cyclic guanosine monophosphate-adenosine monophosphate, KEGG Kyoto Encyclopedia of Genes and Genomes, GO Gene Ontology, LAD left anterior descending coronary artery, ACSL4 acyl-CoA synthetase long-chain family member 4, TFR transferrin receptor, SLC7A11 solute carrier family 7, member 11, GPX4 glutathione peroxidase 4, MDA malondialdehyde

To elucidate the specific pathway through which STING contributes to MI/R injury, RNA sequencing (RNA-seq) was conducted on heart sections from *Sting*-CKO mice and their control mice following I/R (Fig. 3a). Analysis with Kyoto Encyclopedia of Genes and Genomes (KEGG) pathways revealed a significant enrichment of genes involved in cell growth and death pathways among the differentially expressed genes (DEGs) (Fig. 3b). Accordingly, we evaluated the extent of cell death in heart tissues and CMs derived from *cgas*-CKO or *Sting*-CKO mice, as well as their control mice subjected to I/R injury. Remarkably, a significant increase in Tunel-positive cells was discernible in the hearts subsequent to I/R. Conversely, both *cgas*-CKO and *Sting*-CKO mice exhibited notably reduced levels of Tunel-positive cells when compared to their respective control groups (Supplementary Fig. 2a, b, Fig. 3c, Supplementary Fig. 2e, f), suggesting less cell death. Considering that Tunel can also detect DNA breaks caused by necrosis or other cell death processes,^2^ we quantified the expression levels of key cell death markers, such as Pro-caspase-3, Cleaved-caspase3 and B-cell lymphoma-2 (BCL-2), using Western blot assays. The results showed no significant change of protein abundance in *Sting*-CKO mice post-I/R compared with their control mice (Supplementary Fig. 2c, d). Collectively, these results confirm that the absence of STING attenuates cell death in the heart following I/R injury, but not through the modulation of apoptosis.

Since there are many modes of death in cells, according to Gene Ontology (GO) enrichment analysis, the heart sections from *Sting*^fl/fl^ mice exhibited a prominent contribution to autolysosome-related, oxidative stress and ferroptosis signaling pathways following I/R compared to *Sting*-CKO mice (Fig. 3d). Tunel staining is also used as a marker for ferroptosis. We next investigated the effect of STING on the biomarkers of ferroptosis, including lipid peroxidation, reactive oxygen species (ROS) accumulation^17^ and GPX4 degradation, as well as the expression of some ferroptosis-related proteins. Lipid peroxidation has, of late, been recognized as playing a direct role in facilitating necrosis and ferroptosis.^18^ To investigate whether STING modulates ferroptosis triggered by A/R, we assayed the generation of malondialdehyde (MDA), an end product of lipid peroxidation. The results showed that after A/R, STING was highly effective in promoting the production of MDA, comparable to the positive control Erastin (Fig. 3e), meanwhile, STING activation blocked the inhibition of MDA in ferroptosis induced by ferrostatin-1 (Fer-1, a ferroptosis inhibitor). These observations suggest that STING functions upstream of lipid peroxidation, thereby playing a pivotal role in ferroptosis regulation.

Given that mitochondrial damage and oxidative stress response are pivotal events in I/R,^17^ to further test the relationship of STING and oxidative stress in heart, we first confirmed the presence of oxidative stress injury by detecting the generation of ROS following A/R in CMs. Exposure of cultured CMs to A/R triggered an elevation in ROS production,^19^ MPCs were pretreated with Fer-1 for 12 h to attenuate ROS levels before live cell imaging, subsequently, cGAMP was added at 30.36'' to activate STING, and observations and imaging were terminated once ROS levels stabilized (Fig. 3f). Interestingly, the inhibition of ROS in ferroptosis by Fer-1 was significantly impeded in the presence of cGAMP. The results indicated that STING activation leads to an increase in ROS generation.

Having analyzed the impact of STING on ferroptosis biomarkers, encompassing lipid peroxidation and ROS accumulation, we next aimed to confirm the mechanism underlying STING’s promotion of ferroptosis by assessing lipid peroxidation and the abundance of ferroptosis factors. We used Western blot to evaluate the abundance of expression for acyl-CoA synthetase long-chain family member 4 (ACSL4), transferrin receptor (TFR), solute carrier family 7, member 11 (SLC7A11) and GPX4, all of which are established to be protein targets of ferroptosis. We observed decreases in the expression of ACSL4 and TFR, alongside elevations in GPX4 and no significant change in SLC7A11, in *Sting*-CKO mice following I/R compared to their controls (Fig. 3g, h). Up-regulation of ferroptosis was further confirmed at the protein level in MPCs post-A/R (Fig. 3i, j). Interestingly, *Sting* deficiency blocked the degradation of GPX4 during ferroptosis triggered by A/R, while GPX4 degradation is essential for promoting lipid peroxidation in ferroptosis.^20^ Next, we measured the *gpx4* mRNA levels in cardiac tissue from both Sham and I/R-treated mice using qPCR. Our results showed that *gpx4* mRNA levels remained unchanged between the *Sting*^fl/fl^ and *Sting*-CKO groups post-I/R (Supplementary Fig. 2h), suggesting that the reduction in GPX4 protein is not due to transcriptional downregulation but rather to post-translational regulation. Taken together, we conclude that STING is a potent activator of ferroptosis, facilitating GPX4 protein degradation, lipid peroxidation and ROS accumulation. Subsequently, we delved into the molecular occurrences in which I/R-mediated STING was associated with the reduction of GPX4.

### Targeting of GPX4 by STING

**Fig. 4:**
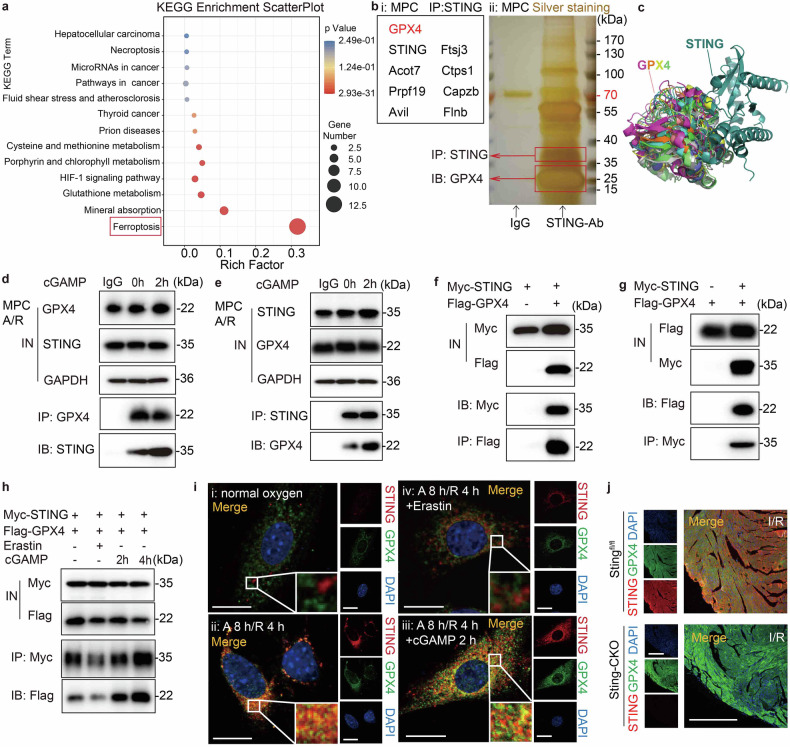
Targeting of STING by GPX4. **a** KEGG displayed ferroptosis pathway enrichment. **b** Silver staining and LC-MS/MS analysis for tandem affinity purification using STING protein affinity antibodies in MPCs post-A/R. **c** Images showing ten potential contact modes according to molecular-docking results between STING (PDB ID: 4F5W) and GPX4 (PDB ID: 5L71). **d** Co-IP assay of MPCs to examine whether endogenous GPX4 interacts with STING and this combination is influenced by short point-stimulation of cGAMP. IB: STING, IP: GPX4. **e** Co-IP assay of MPCs to examine whether endogenous STING interacts with GPX4 and this combination is influenced by short point-stimulation of cGAMP. IB: GPX4, IP: STING. **f** Co-IP assay of HeLa cells co transfected with Flag-GPX4 and Myc-STING to examine whether STING interacts with GPX4. IB: Myc, IP: Flag. **g** Co-IP assay of HeLa cells co transfected with Flag-GPX4 and Myc-STING to examine whether GPX4 interacts with STING. IB: Flag, IP: Myc. **h** Co-IP assay of HeLa cells co transfected with Flag-GPX4 and Myc-STING under Erastin or cGAMP delivering to examine whether GPX4 and STING interaction is influenced by cGAMP time-grant or Erastin. IB: Flag, IP: Myc. **i** Double immunofluorescence analysis for detecting STING and GPX4 and their co-localisation in HeLa cells co transfected with Myc-STING and Flag-GPX4 under cGAMP or Erastin addition. The positive reaction for the Myc-label is shown in red, and that for the Flag-label is shown in green. The positive reaction for co-localisation is displayed in yellow. Scale bar = 20 μm. **j** Double immunofluorescence analysis for detecting GPX4 and STING in the same heart section of the *Sting*^fl/fl^ or *Sting*-CKO border region. The positive reactions of tissue sections are displayed in green (GPX4) and red (STING). The positive reaction for co-localisation is displayed in yellow. Scale bar = 200 μm. LC liquid chromatography, MS mass spectrometry, PDB Protein Data Bank, Co-IP co-immunoprecipitation, IN input, IB immunoblotting, IP immunoprecipitation, Flag-GPX4 Flag-labeled GPX4 plasmid, Myc-STING Myc-labeled STING plasmid

The upregulation of the ferroptosis program following STING activation hinted at the potential for MI/R injury to promote unanticipated activation of a signal-transduction chain cascade involving STING. We sought to identify potential gene factors and explore the feasibility of leveraging them to mitigate the exacerbation of MI/R injury in STING-activated mice. Firstly, we analyzed the KEGG pathways expressed in DEGs identified through RNA-seq. (Fig. 3a). Remarkably, ferroptosis-related genes were significantly enriched among these DEGs (Fig. 4a). Next, tandem affinity purification was performed in MPCs using STING protein affinity antibodies to determine the molecular mechanism by which STING regulates ferroptosis. Silver staining of the immune purification materials (Fig. 4b i) and subsequent analysis using LC-MS/MS (Fig. 4bii) were employed to identify the STING protein chaperone. This method revealed several proteins, particularly the ferroptosis factor GPX4. We used PyMOL to display the predicted results of molecular docking analysis between STING (Protein Data Bank [PDB] ID: 4F5W) and GPX4 (PDB ID: 5L71). Figure 4c depicts ten potential binding modes, with a Match-Align score of 8.157, increasing the likelihood that STING may directly interact with GPX4.

To further corroborate the direct interaction between STING and GPX4, we employed a STING-specific antibody to pull down its protein chaperone, then combined this protein purification products with GPX4 antibody and monitored them in immunoprecipitation (IP) assays. The endogenous co-IP experiments revealed that endogenous STING and GPX4 formed a complex in CMs post-A/R, with cGAMP further enhancing the formation of this complex (Fig. 4d). Correspondingly, STING was detectable in the endogenous purified products of GPX4 (Fig. 4e), indicating a direct interaction between these two proteins. Next, we expressed Myc-tagged STING (Myc-STING) and Flag-tagged GPX4 (Flag-GPX4) plasmids in HeLa cells that stably overexpressed these constructs. Myc or Flag IP was then performed to investigate the interaction between Myc-STING and Flag-GPX4. We used Western blot assays to analyze the input and eluate, and the results showed that Myc-STING enrichment could be detected in the purified product eluted by excessive Flag peptide of Flag-GPX4 (Fig. 4f). Correspondingly, Flag-GPX4 enrichment was also observable in the purified product of Myc-STING (Fig. 4g). Furthermore, we found that the interaction between GPX4 and STING was augmented in a time-dependent manner in HeLa cells treated with cGAMP (Fig. 4h), while Erastin diminished the strength of the interaction.

To confirm the interaction between STING and GPX4, immunostaining was performed to analyze the co-localization of STING and GPX4 in MPCs. Under normoxic conditions, MPCs exhibited low expression of STING and no co-localization with GPX4 (Fig. 4ii). Following acute A/R, STING was activated and formed aggregate dots with GPX4 (Fig. 4i ii), while the two had massive aggregation and co-localization with cGAMP stimulation (Fig. 4i iii). Interestingly, upon Erastin, GPX4 had few co-localization with STING (Fig. 4i iv). These findings align closely with the experimental results obtained from the Co-IP study. Together, these evidence strongly indicates that STING and GPX4 interact directly in CMs post-A/R. Furthermore, with increasing STING activation by cGAMP, a more pronounced cluster distribution of STING and GPX4 is observed, indicating an enhanced binding effect between the two proteins.

Given the known co-localization and interaction between STING and GPX4 in both mouse and human cells, coupled with the previously mentioned role of STING in regulating ferroptosis, we surmised that GPX4 might be involved in modulating I/R injury which STING contribute to. Immunostaining analysis of heart slices from *Sting*^fl/fl^ and *Sting*-CKO mice highlighted the I/R-induced enhancement of STING expression and its co-localization with GPX4 in I/R border region of *Sting*^fl/fl^ hearts (Fig. 4j). Conversely, GPX4 became much brighter and had no co-localization with STING when *Sting* was specifically deleted. These results further confirmed the previously discovered effect of STING on inducing ferroptosis and the binding between STING and GPX4 post-I/R. It is noteworthy to mention that this binding has the potential to elicit a downregulation in GPX4 expression, necessitating further investigative efforts.

In summary, a direct interaction occurs between STING and GPX4 and the STING activator cGAMP mediates the crosstalk between GPX4 and STING. This effect is conserved in mouse and human cell lines. As the absence of *Sting* actually led to an increased expression in GPX4 in heart slices following I/R, which aligns with previous findings indicating that STING can induce ferroptosis. Based on these observations, we speculate that STING may directly bind to GPX4, thereby inhibiting its activity and ultimately leading to ferroptosis.

### STING and GPX4 directly interact at the amino acid residues N146 of GPX4 and T267 of STING

**Fig. 5:**
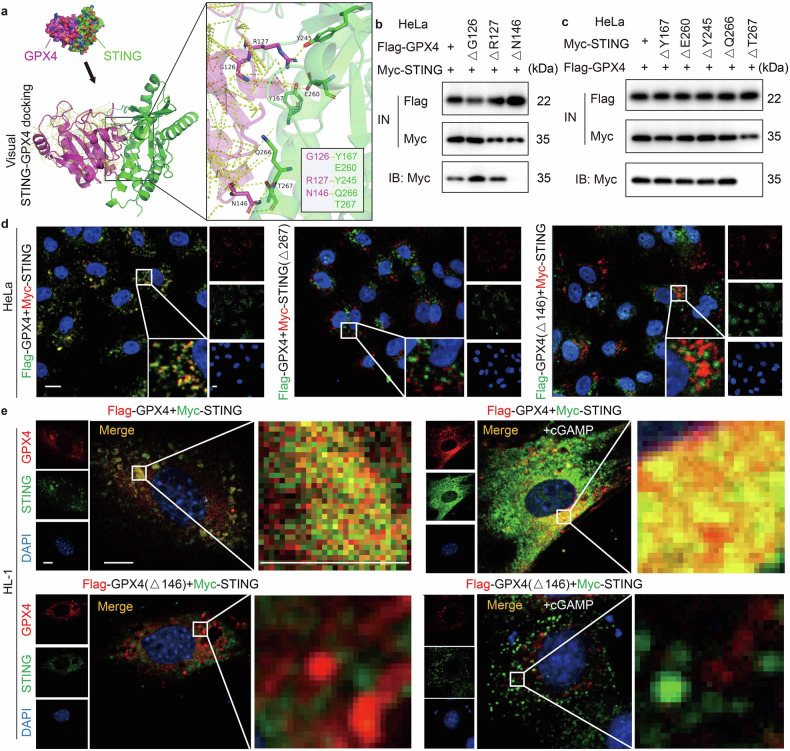
STING and GPX4 directly interact at the amino acid residues N146 of GPX4 and T267 of STING. **a** Representative images of molecular docking results to show the potential contact sites between GPX4 and STING. **b** Co-IP assay of HeLa cells co-transfected with Flag-GPX4 or Flag-GPX4 point mutation (Flag-GPX4[Δ*G*126], Flag-GPX4[Δ*R*127] or Flag-GPX4[Δ*N*146]) and Myc-STING, to examine the potential contact site between GPX4 and STING. IB: Myc, IP: Flag. **c** Co-IP assay of HeLa cells co-transfected with Flag-GPX4 and Myc-STING or Myc-STING point mutation (Myc-STING[Δ*Y*167], Myc-STING[Δ*E*260], Myc-STING[Δ*Y*245], Myc-STING[Δ*Q*266] or Myc-STING[Δ*T*267]) to examine the potential contact site between GPX4 and STING. IB: Myc, IP: Flag. **d** Double immunofluorescence analysis for detecting STING and GPX4 and their co-localisation in HeLa cells co-transfected with Myc-STING and Flag-GPX4, Flag-GPX4 and Myc-STING[Δ*T*267] or Myc-STING and Flag-GPX4[Δ*N*146]. The positive reaction for the Myc-label is shown in red, and that for the Flag-label is shown in green. The positive reaction for co-localisation is displayed in yellow. Scale bar = 20 μm. **e** Double immunofluorescence analysis for detecting STING and GPX4 and their co-localisation in HL-1 cells co-transfected with Myc-STING and Flag-GPX4 or Myc-STING and Flag-GPX4 [Δ*N*146] under cGAMP addition or not. The positive reaction for the Myc-label is shown in green, and that for the Flag-label is shown in red. The positive reaction for co-localisation is displayed in yellow. Scale bar = 5 μm. Scale bar of the enlarged image = 2.5 μm. G Gly, R Arg, N Asn, Y Tyr, E Glu, Q Gln, T Thr

To further elucidate the binding site where STING associates with GPX4, we conducted a meticulous examination of PDB graphics and associated data. Apo STING (non-ligand bound) oligomer (PDB ID: 4F5W) and GPX4 (PDB ID: 5L71) were selected for protein interaction prediction. The prediction results revealed that STING might engage in protein docking with GPX4 at the amino acid positions of *Y*167, *E*260, *Y*245, *Q*266, or *T*267. Correspondingly, GPX4 could potentially engage in protein docking with STING at the amino acid positions of *G*126, *R*127, or *N*146 (Fig. 5a). Based on the aforementioned predictions, IP experiments were conducted on HeLa cells expressing Flag-GPX4 mutants and Myc-STING mutants to determine the interaction domain between STING and GPX4. Initially, three GPX4 peptide segments, Δ*G*126, Δ*R*127 and Δ*N*146, were evaluated. With the analysis of the Western blot results, we found that only Flag-GPX4-∆*N*146 mutant lost its capacity to interact with STING, indicating a critical role of this amino acid in the interaction between the two proteins (Fig. 5b). On the other hand, Myc-tagged STING mutants with specific amino acid mutations, namely Myc-STING-Δ*Y*167, Myc-STING-Δ*E*260, Myc-STING-Δ*Y*245, Myc-STING-Δ*Q*266 and Myc-STING-Δ*T*267, were constructed. The results demonstrated that the absence of the *T*267 amino acid in STING abolished the interaction between STING and GPX4 (Fig. 5c).

Furthermore, in HeLa cells stably overexpressing Myc-STING and Flag-GPX4, we observed that GPX4 lost its co-localization with STING when the *T*267 site of STING was absent (Fig. 5d), even if the entire genome of GPX4 was overexpressed. Similarly, the interaction between STING and GPX4 disappeared when GPX4 lost its *N*146 site (Fig. 5d). Collectively, these data conclusively demonstrate that STING physically interacts with GPX4-*N*146 through the *T*267 amino acid of STING.

Utilizing super-resolution imaging microscopy (SIM) devices to enhance the clarity of immunostaining images, we observed robust binding and co-localization between STING and GPX4 in HL-1 cells transfected with Myc-tagged STING and Flag-tagged GPX4 plasmids. Employing this approach, we were able to achieve a more precise comprehension regarding the mode of interaction between these two proteins within the cellular context (Fig. 5e). As depicted in Fig. 4h, cGAMP delivery further accelerated STING-GPX4 complex formation. Notably, when Flag-GPX4-Δ*N*146 and Myc-STING were simultaneously transfected, GPX4 lost its co-localization with STING. At this point, even upon the delivery of cGAMP, the co-localization between the two proteins could not be restored, thus highlighting the pivotal role of this amino acid in facilitating the interaction between these two proteins.

### STING promotes ferroptosis via autophagy-lysosome-mediated degradation of GPX4

**Fig. 6:**
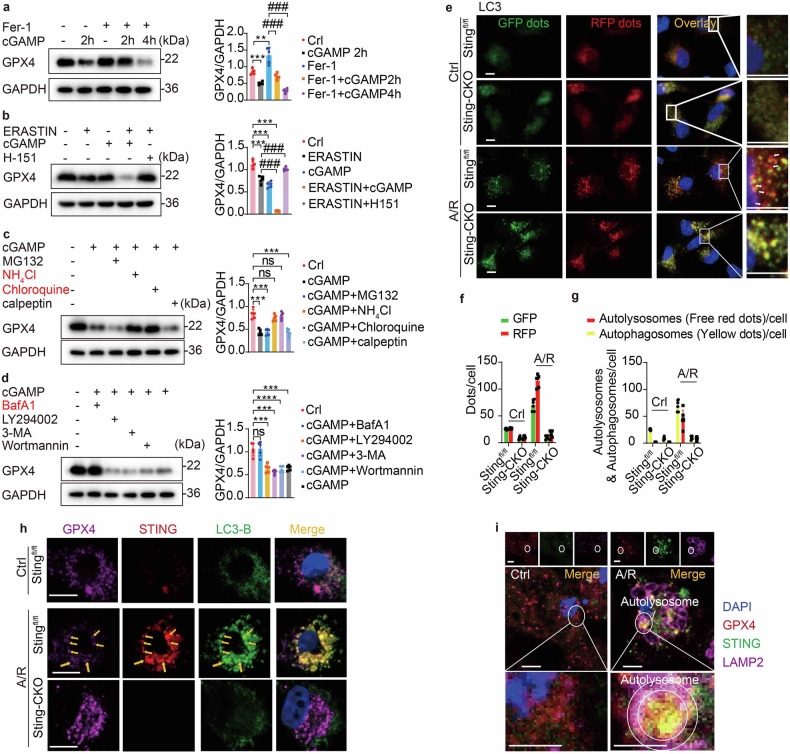
STING promotes ferroptosis via autophagy-lysosome-mediated degradation of GPX4. **a** Western blot and quantification of GPX4 expression in MPCs influenced by Fer-1 or cGAMP (*n* = 5). **b** Western blot and quantification of GPX4 degradation in MPCs induced by Erastin, cGAMP or H-151 (*n* = 5). **c** Western blot and quantification of GPX4 degradation in MPCs induced by cGAMP and its blocking using MG-132, NH_4_Cl, chloroquine and calpeptin (*n* = 5). **d** Western blot and quantification of GPX4 degradation in MPCs induced by cGAMP and its blocking using Baf A-1, LY294002, 3-MA and Wortmannin (*n* = 5). **e**–**g** Immunofluorescence analysis for autophagic flow presented by mRFP-GFP-LC3 in *Sting*^fl/fl^ or *Sting*-CKO MPCs post-A/R or not. The positive reactions for autophagy are displayed in red dots. Scale bar = 10 μm. **h** Triple immunofluorescence analysis for detecting GPX4, STING, and LC3B in *Sting*^fl/fl^ or *Sting*-CKO MPCs. The positive reactions for co-localisation are displayed in yellow (LC3B and STING co-localisation) or pink (co-localisation of the three). Scale bar = 10 μm. **i** Triple immunofluorescence analysis for detecting GPX4, STING, and LAMP2B in MPCs post A/R or not. The positive reactions for co-localisation are displayed in yellow (GPX4 and STING co-localisation) or pink (co-localisation of the three). Scale bar = 10 μm. Mean ± SEM, NS non-significant, **P < 0.01, ***P < 0.001, ****P < 0.0001 and ### *P* < 0.001. Baf A-1 Bafilomycin A1, LAMP2B lysosomal-associated membrane protein 2B

The degradation of GPX4 protein is a pivotal event in ferroptosis, ROS generation and irreversible lipid peroxidation, ultimately resulting in cell death. ^21^ In Fig. 4j, we also observe the potential degradation effect of STING on GPX4 within the myocardial tissue. Next, we verified the impact of STING on GPX4 degradation during ferroptosis. Given our observation that *Sting* deficiency blocked the degradation of GPX4 during ferroptosis induced by I/R or A/R, we further explored the stimulatory effect of the STING activator, cGAMP, on GPX4 degradation. Notably, we observed that cGAMP resulted in a decrease in the levels of GPX4 protein, contrary to the negative control Fer-1 (Fig. 6a). Treating MPCs with the ferroptosis inhibitor Fer-1 led to an elevation of GPX4 protein levels, but this elevation was blocked in the presence of cGAMP. Additionally, GPX4 activity can be suppressed by Erastin, which shares similar effects to cGAMP as previously mentioned, leading to an accumulation of intracellular lipid peroxides. To evaluate whether cGAMP enhances the degradation of GPX4, we utilized Erastin in our experiments. Administration of cGAMP indeed initiated the degradation of GPX4 (Fig. 6b). Furthermore, the addition of H-151 (a STING inhibitor) was also able to protect ferroptosis and inhibit the degradation of GPX4 induced by Erastin. Taken together, STING activation could elicit the degradation of GPX4.

Subsequently, we delved into the underlying mechanism governing the regulation of GPX4 degradation by STING. Protein degradation post-transcriptionally occurs primarily through two mechanisms: ubiquitin-mediated proteasomal degradation and autophagy-mediated lysosomal degradation.^22^ The findings presented in Fig. 3d also demonstrate the enrichment of autolysosomal pathways in *Sting*^fl/fl^ mice during I/R injury, suggesting that STING may exert regulatory influence on the degradation of GPX4 via the autophagic pathway. Therefore, we employed several protein degradation inhibitors, including proteasomal inhibitors (MG-132 and calpeptin) and lysosome inhibitors (chloroquine (CQ) and NH_4_Cl) to specifically block these degradation pathways. Interestingly, only the application of the lysosome inhibitors CQ or NH_4_Cl could effectively block GPX4 degradation induced by cGAMP and contributed to GPX4 abundance (Fig. 6c), indicating their participation in the modulation process of GPX4 homeostasis during A/R exposure. Bafilomycin A1(Baf A-1) serves as a late-stage inhibitor of autophagy, effectively preventing the maturation of autophagic vesicles by disrupting the fusion process between autophagosomes and lysosomes. LY294002, 3-MA and Wortmannin act as inhibitors targeting the initial stage of autophagy, effectively blocking the formation of autophagosomes. Through the analysis of Western blot assays, we discovered that only Baf A-1 was capable of blocking GPX4 degradation induced by cGAMP (Fig. 6d), indicating that STING facilitated the autophagic degradation of GPX4 by promoting the fusion of autophagosomes and lysosomes.

Autophagy, a pivotal cellular process responsible for the degradation of proteins,^23^ emerged as a significant potential factor mediating the reduction of GPX4, which was induced by STING subsequent to I/R. As shown in supplementary Fig. 2g, STING deletion could suppress autophagy that was triggered by I/R. In order to further corroborate the intervention of STING in the autophagic degradation of GPX4 through its modulation of autophagosome-lysosome binding, we employed the RFP-GFP-LC3 dual fluorescence autophagy indicator system for meticulously labeling and tracking alterations in microtubule-associated proteins 1A/1B light chain 3B (LC3B) and autophagic flux. Initial observations revealed a robust activation of autophagy under A/R conditions (Fig. 6e–g). The chimeric fluorescent protein-LC3 firmly anchored itself to the autophagosome membrane and merged with the lysosome to form autolysosomes. Notably, during this process, GFP fluorescence waned, signifying the seamless transition from autophagosomes to autolysosomes. However, in *Sting*-CKO MPCs, a remarkable increase in yellow fluorescent dots was observed, with less red dots. This phenomenon robustly corroborates our Western blot findings, indicating a hindrance in the fusion of autophagosomes and lysosomes, as well as a disruption in the maturation process of autophagosomes. In essence, the absence of Sting prevents the elimination of GPX4 by impeding the union of autophagosomes and lysosomes.

STING is recognized for its ability to initiate autophagy through the lipidation of LC3B and the subsequent formation of autophagosomes, independently of TBK1 activation and interferon induction.^13^ We next examined the effect of STING, LC3B and GPX4 in ferroptosis. Utilizing tricolor immunofluorescence analysis, we observed that A/R conditions activated STING, resulting in the formation of LC3 puncta in *Sting*^fl/fl^ MPCs but not *Sting*-deficient cells (Fig. 6h). This finding concurs with previously reported literature results.^13^ The clear co-localization of LC3 puncta with both STING and GPX4 was observed in *Sting*^fl/fl^ MPCs but not in cells that lack STING, suggesting GPX4 was targeted by STING-induced autophagy post-A/R. To localize the interaction of STING and GPX4, we co-stained for STING, GPX4, and markers of endoplasmic reticulum-Golgi intermediate compartment (ERGIC), coat protein I (COP-I), and LC3 in MPCs. Our results demonstrated that the STING-GPX4 complex initially localizes to the ERGIC. The STING-GPX4 complex is subsequently trafficked towards the ERGIC, vesicles of COP-I, and autophagosomes of LC3 (Supplementary Fig. 3).

Additionally, lysosomal-associated membrane protein 2B (LAMP2B) mediates the mechanism of autophagosome-lysosomal fusion.^24^ A/R-stimulated autophagosome formation was visualized by LAMP2B. Imaging revealed that the GPX4-STING puncta induced by A/R was enclosed by the LAMP2B-labeled autolysosome membrane ring (Fig. 6i). This suggests that the interaction between STING and GPX4 occurs in the ERGIC and that STING facilitates the recruitment of GPX4 into autophagosomes for subsequent autophagic degradation. These findings suggest that autophagy induced by STING facilitated the elimination of GPX4 in CMs post-A/R.

### AAV-mediated GPX4 therapy shields cardiac function against the severe I/R injury triggered by STING activation

**Fig. 7:**
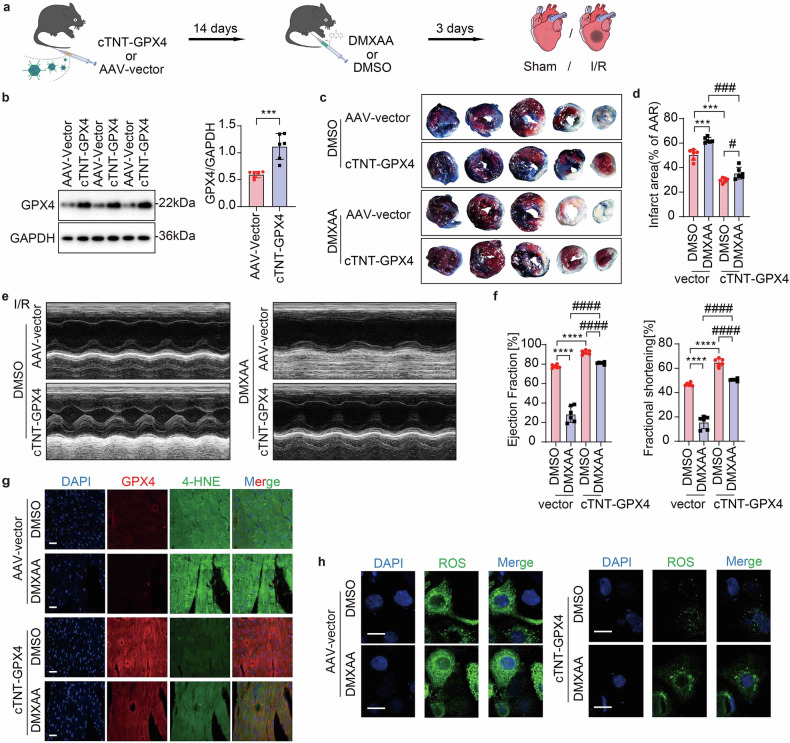
AAV-mediated GPX4 therapy shields cardiac function against the severe I/R injury triggered by STING activation. Effect of AAV-*cTNT*-GPX4 on heart dysfunction therapy in *C57BL/6J* mice with DMXAA or DMSO addition post-I/R: **a** Schematic diagram depicting the time course of I/R-induced cardiac dysfunction receiving AAV or DMXAA. **b** Western blot of verifying successful overexpression of GPX4 in CMs (*n* = 6). **c**, **d** Myocardial infarct size (% of AAR) with representative tissue sectioning (*n* = 6). **e**, **f** Echocardiography and measured EF% and FS% (*n* = 6). **g** Double immunofluorescence analysis for GPX4 and 4-HNE in the same heart section of the border region. Scale bar = 20 μm. **h** Immunofluorescence imaging analysis for detecting ROS in AAV-*cTNT*-GPX4 or AAV-Control infection mice CMs under DMSO or DMXAA addition following A/R. Scale bar = 10 μm. Mean ± SEM, NS, non-significant, **P < 0.01, ***P < 0.001, ****P < 0.0001, #*P* < 0.05 and ###*P* < 0.001. AAV adeno-associated virus, 4-HNE 4-Hydroxynonenal, cTNT-GPX4 AAV-Mus-GPX4-*cTNT*-*C*-GFP, DMXAA Vadimezan

Given our findings, we subsequently verified that GPX4 functions as a downstream factor of STING, and further delved into the potential of GPX4 as a therapeutic target for STING-associated I/R injury. An adeno-associated virus (AAV) targeting GPX4 (AAV-GPX4) driven by the *cTNT* promoter in CMs and labeled with GFP (referred to *cTNT* -GPX4) was generated to examine whether the overexpression of GPX4 can alleviate the progressive decline of cardiac function in STING-activated mice under in-vivo conditions. AAV-GPX4-GFP was administered through tail-vein injection 14 days before the induction of I/R. Subsequently, intraperitoneal injection of DMXAA (a STING activator) or DMSO was carried out every day for three days before I/R operation (Fig. 7a). Prior to model establishment, the overexpression efficiency of GPX4 in CMs isolated from three or six mice was confirmed using Western blot and immunofluorescence staining (Fig. 7b, Supplementary Fig. 4a). Delivering DMXAA could increase the infarct size, while GPX4 overexpression minimized the infarct size in STING-activated mice (Fig. 7c, d), suggesting a protective role of GPX4 overexpression against I/R injury induced by STING activation. Echocardiographic analysis revealed that STING activation indeed exacerbated cardiac dysfunction following I/R, however, GPX4 overexpression significantly ameliorated the deteriorating cardiac function in STING-activated mice upon DMXAA administration, as evidenced by increased EF and FS post-I/R (Fig. 7e, f).

4-Hydroxy-2-nonenal (4-HNE), which is a widely acknowledged byproduct emerging from lipid peroxidation, functions as a crucial indicator of ferroptosis.^25^ In our results, STING activation led to a significant elevation in 4-HNE levels, indicating increased lipid peroxidation (Fig. 7g), while GPX4 overexpression was able to reverse this increase in lipid peroxidation caused by DMXAA, suggesting a downregulation of ferroptosis. The in vitro detection of ROS accumulation levels in MPCs robustly corroborated this observation (Fig. 7h). To delve deeper into the impact of the cGAS-STING-GPX4 axis on mitochondria, we conducted several experiments. These experiments encompassed measurements of the ATP level, assessment of the mitochondrial membrane potential (ΔΨm), and determination of the oxygen consumption rate (OCR) to assess mitochondrial health, ATP content, membrane potential, and respiratory capacity under A/R conditions. The results showed that, blocking cGAS/STING signaling or overexpressing GPX4 partially restored ATP levels, ΔΨm, basal mitochondrial respiration, ATP production and both maximal and spare respiratory capacities (Supplementary Fig. 4b–g, Supplementary Fig. 5a–c), signifying the partial rejuvenation of mitochondrial function induced by cGAS/STING deletion or GPX4 overexpression.

Above all, it can be concluded that, despite the potentiation of I/R damage in STING-overexpressing mice, the upregulation of GPX4 retains a remarkable capacity to prevent lipid peroxidation and ferroptosis, thereby safeguarding cardiac function. In conclusion, cGAS/STING deletion or GPX4 overexpression protects against deteriorating cardiac dysfunction and mitochondrial damage following I/R in STING-activated mice, pointing towards a potential therapeutic strategy to alleviate the adverse effects of STING overexpression on cardiac function.

### STING is a potential therapeutic target to inhibit cardiac dysfunction post-I/R

**Fig. 8:**
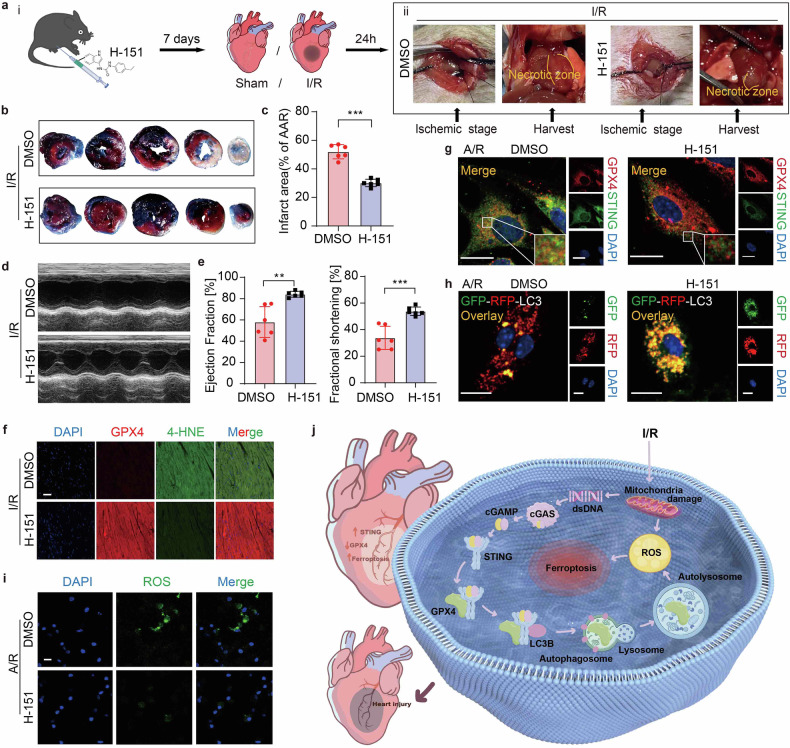
STING is a potential therapeutic target to alleviate cardiac dysfunction post-I/R. Effect of H-151 on heart dysfunction in *C57BL/6J* mice post-I/R: **a** i. Schematic diagram depicting the time course of I/R-induced cardiac dysfunction receiving H-151 or DMSO. ii. Representative photographic images of hearts with DMSO or H-151 addition post-I/R. **b**, **c** Myocardial infarct size (% of AAR) with representative tissue sectioning (*n* = 6). **d**, **e** Echocardiography and measured EF% and FS% (*n* = 6). **f** Double immunofluorescence analysis for GPX4 and 4-HNE in the same heart section of the border region. Scale bar = 50 μm. **g** Immunofluorescence analysis for GPX4 and STING in DMSO or H-151 addition mice CMs post A/R. Scale bar = 10 μm. **h** Immunofluorescence analysis for autophagic flux presented by mRFP-GFP-LC3 in DMSO or H-151 addition mice CMs post A/R. The positive reactions for autophagy are displayed in red dots. Scale bar = 20 μm. **i** Immunofluorescence imaging analysis for detecting ROS in DMSO or H-151 addition mice CMs post A/R. Scale bar = 20 μm. **j** Schematic diagram showing the mechanism of STING-promoted MI/R injury. Part of the image was drawn by Figdraw. Mean ± SEM, NS non-significant, **P < 0.01, ***P < 0.001, and ****P < 0.0001

To delve deeper into I/R treatment strategies that harness the STING-GPX4 signaling pathway, we selected H-151 to evaluate its potential in alleviating the harmful impacts of I/R on cardiac function and to identify potential therapeutic agents for the treatment of I/R injury. Prior to I/R surgery, mice were intraperitoneally administered with H-151 every two days for a total duration of 7 days (Fig. 8a i). Concurrently, the degree of myocardial necrosis was clearly discernible in the in vivo cardiac images (Fig. 8a ii), demonstrating the effective control exerted by H-151 on the extent of necrosis within the cardiac functional area. Notably, in the presence of H-151, a reduction in infarct size (Fig. 8b, c) and restoration of heart function (Fig. 8d, e) were observed. Furthermore, H-151 addition was able to alleviate the increase of 4-HNE caused by I/R, suggesting a downregulation of lipid peroxidation and ferroptosis (Fig. 8f).

Subsequently, we delved into the pharmacological mechanism underlying the ameliorative effects of H-151 on myocardial injury following I/R in vitro. In our results, H-151 impeded the interaction between STING and GPX4 (Fig. 8g), blocked the autophagic flux (Fig. 8h), thus minimized ROS accumulation (Fig. 8i) to mitigate myocardial ferroptosis.

The effect of H-151 highlights its potential in reducing the area of I/R injury and preserving cardiac function. Taken together, STING emerges as a promising therapeutic target to attenuate the cardiac impairment that occurs post-I/R, with its deleterious effects being modulated through the regulation of GPX4, suggesting a promising avenue for the intervention of cardiac remodeling.

To demonstrate the clinical significance of this study and to investigate the presence of the STING-GPX4 signaling axis in patients suffering from ischemic heart disease, we conducted measurements of serum proteins in patients both before and after percutaneous coronary intervention (PCI). The baseline characteristics of the patients involved in this study are outlined in Supplementary Table S3. Our results revealed that following PCI, there was an elevation in the expression level of STING protein in the patients’ serum, accompanied by a decrease in the expression level of GPX4 (Supplementary Fig. 5d). This finding suggests that the STING-GPX4 axis might play a crucial role in patients experiencing I/R. Additionally, we observed an increased expression in STING protein and a reduction in GPX4 protein content in the lysate of CMs that were derived from human embryonic stem cells, which were induced by A/R conditions (Supplementary Fig. 5e). These results further underscore the potential significant biological role of the STING-GPX4 axis in the human heart.

## Discussion

Ferroptosis occurs during myocardial ischemia-reperfusion (MI/R) injury and is accompanied by the degradation of GPX4. Here, we provide multiple lines of evidence that STING plays an initiating role in MI/R-induced myocardial ferroptosis by directly binding to GPX4 and triggering autophagy-lysosome-mediated GPX4 degradation. I/R elevates cytoplasmic DNA, activating dsDNA-cGAS-STING signaling in cardiomyocytes (CMs). Deletion of cGAS or STING in CMs reduces oxidative stress, ferroptosis, and MI/R injury, while STING activation exacerbates them. Mechanistically, STING interacts with GPX4 at specific residues (N146 of GPX4 and T267 of STING) to initiate autophagy and lysosomal degradation. STING forms a positive feedback loop via the dsDNA-cGAS-STING-GPX4 axis, exacerbating ferroptosis. Two treatment strategies for MI/R injury-AAV-mediated GPX4 overexpression and STING antagonists-show promise for preventing and reducing I/R injury, highlighting potential clinical targets. Collectively, our findings revealed that the accumulation of STING during MI/R induces ferroptosis by interfering with GPX4.

One effective cardioprotective approach in myocardial disorders is to prevent the death of cardiac cells.^1^ Recent studies have shown that cGAS-STING immuno-signaling pathway plays a role in various ischemic diseases, encompassing cerebral, renal, enteral, and hepatic I/R scenarios.^26–34^ Furthermore, cGAS has been implicated as a player in the inflammatory response observed in myocardial infarction (MI).^27^ IRF3, a downstream signaling molecule of the cGAS-STING pathway, is recognized for its role in the pathological process following MI.^31^ To date, major research efforts regarding the cGAS-STING pathway post-MI have focused on individual cytokines, such as interferon (IFN),^35^ or macrophage immunity.^27^ There remains a gap in understanding the direct functional relevance of cGAS-STING in MI/R injury of the heart and the progression of ferroptotic damage. In the present research, we confirmed that STING induced ferroptosis in CMs during MI/R injury.

The I/R process is characterized by an initial phase of ischemia and hypoxia, followed by a reperfusion stage characterized by the re-establishment of blood flow. Notably, this reperfusion phase has the most substantial and harmful damage associated with I/R, encompassing metabolic reprogramming, DNA impairment, the enhanced expression of pro-inflammatory genes, and mitochondrial malfunction. Understanding the intricate mechanisms underlying these pathological processes is crucial for developing effective therapeutic strategies to mitigate the harmful effects of I/R.^1,2^ Studies have reported that circulating levels of DNA were significantly elevated following cardiac ischemia.^36^ We further confirmed that during I/R injury, there occurs a profound degree of DNA damage, resulting in the leakage of DNA into the cytosol, subsequently triggering the upregulation of the cGAS-STING signaling pathway in CMs rather than fibroblasts or macrophages during acute I/R injury. Besides, our study found the highest cGAS-STING signaling activation in the border region, compared to scar or non-ischemic region, aligning with the fact that it is the most dynamic area with active oxidative stress and cell death pathways, including ferroptosis. Additionally, STING appears to be an indispensable downstream factor for cGAS to execute its functional role in the context of I/R.

Since the role of cGAS and STING in CMs is poorly understood, we generated CM-specific *cgas*-knockout (*cgas*^fl/fl^ *Myh6*^cre^ [*cgas*-CKO]), STING-knockout (*Sting*^fl/fl^ *Myh6*^cre^ [*Sting*-CKO]) mice and their control mice to investigate the functional importance of CM-specific cGAS and STING in I/R injury. To avoid aging-related subcellular and molecular impacts on CMs and their cardioprotective mechanisms against I/R injury, we used 8-week-old mice for modeling.^37^ Considering the negligible gender impact on the I/R model, we opted for male mice.^38,39^ But we still have shortcomings in model establishment. While mice remain a widely accepted model for studying molecular mechanisms, we recognize the need to validate key findings in larger animal models or human-derived systems in future studies to improve translational relevance.^40^

Because our focus on acute I/R injury and the expression of molecular mediators of inflammation and cellular infiltration needed to be investigated during the first 72 h,^41^ we selected the critical time point of 24 h after reperfusion for animal experiments. However, infarction healing was incomplete at this time.^41^ To assess the effects of cGAS and STING deficiency on I/R injury-induced cardiac fibrosis, we extended reperfusion to 7 days. These findings suggest that cGAS and STING deletion offer cardioprotection beyond the acute phase, mitigating fibrosis and enhancing healing, highlighting the crucial role of the cGAS-STING in post-I/R injury and repair.

As CMs are devoid of immune capabilities, ZJ Chen and other scientists elucidated that STING regulates autophagy, which serves as a fundamental cellular function that operates independently of its canonical immune signaling pathway. Consequently, our primary focus in the context of CM is to investigate the non-immune pathway mediated by STING. I/R injury is a complex pathological process characterized by multiple forms of cell death, such as necrosis, apoptosis, necroptosis, ferroptosis, and autophagy, and the contributions of each mechanism require further discussion.^2^ In our study, we focused on ferroptosis as a significant contributor to infarct size reduction based on its well-established role in ischemic injury, particularly through mechanisms of lipid peroxidation and iron-dependent oxidative damage. Certainly, the cell death mechanisms during I/R injury intertwine. Our research focused solely on the regulatory effect of STING on ferroptosis, which has limitations, especially the lack of analysis of factors related to programmed cell necrosis and pyroptosis. Further exploration of other cell death mechanisms during I/R is warranted in the future.

Although Tunel-positive cells represent a combination of different cell death pathways, the findings from our RNA sequencing (RNA-seq), based on Sting-CKO and their control hearts post-I/R, were noteworthy. These results revealed a novel aspect of MI/R pathophysiology, characterized by the regulation of autolysosomes alongside ferroptosis. Upon I/R insult, a significant enrichment of pathways regulating ferroptosis became highly evident in *Sting*^fl/fl^ mice compared to *Sting*-CKO mice. This offers strong evidence supporting the concept that ferroptosis, a form of cell death reliant on iron, plays a pivotal role in MI/R injury regulated by STING. The degradation of GPX4 protein is a pivotal event in ferroptosis, which in turn prompts mitochondrial damage and the generation of reactive oxygen species (ROS),^17^ leads to irreversible lipid peroxidation, and, consequently, cell death.^21^ We next confirmed the effect of STING on the biomarkers of ferroptosis, including ROS accumulation, lipid peroxidation and GPX4 degradation, as well as the expression of some ferroptosis-related proteins (acyl-CoA synthetase long-chain family member 4, transferrin receptor and solute carrier family 7, member 11). Based on the evidence, we have elucidated that the regulatory impact of STING on I/R injury is not mediated through cell apoptosis or other programmed cell death mechanisms, but rather through the induction of cell ferroptosis. In summary, our research outcomes indicate that STING serves as a potent activator of ferroptosis, effectively promoting the degradation of GPX4, lipid peroxidation and ROS accumulation. STING amplifies myocardial ferroptosis through modulation of oxidative stress injury.

Recent studies report that GPX4 promotes the activation of STING through the maintenance of lipid redox homeostasis.^42^ However, the direct influence of STING on GPX4 and its underlying mechanisms in ferroptosis triggered by MI/R have remained elusive and unexplored. Our findings provide insights into the complex regulatory mechanisms governing the function of GPX4 and STING in the course of I/R events. Thus, our discoveries contribute to augmenting the existing knowledge framework regarding the tissue-level regulation of ferroptosis during MI/R.

Widespread use of agonists and inhibitors enhances our understanding of STING’s direct degradation effect on GPX4. As protein degradation post-transcriptionally occurs primarily through two mechanisms: ubiquitin-mediated proteasomal degradation and autophagy-dependent lysosomal degradation, ^22^ we employed various protein degradation inhibitors (MG-132, calpeptin, chloroquine, NH_4_Cl, Bafilomycin A1, LY294002, 3-MA and Wortmannin) to disturb GPX4 degradation induced by cGAMP. STING is recognized for its ability to initiate autophagy through the lipidation of microtubule-associated proteins 1A/1B light chain 3B (LC3B) and the subsequent formation of autophagosomes, independently of TBK1 activation and interferon induction.^13^ We performed immunofluorescence co-localization analysis of GPX4, STING, endoplasmic reticulum-Golgi intermediate compartment (ERGIC), coat protein I (COP-1), LC3B, and lysosomal-associated membrane protein 2, along with tracking the autophagic flux using GFP-RFP-LC3 in *Sting*-CKO and control CMs. We discovered that cGAMP induces STING trafficking to the ERGIC upon I/R. At the ERGIC, STING binds to GPX4 to form the STING-GPX4 complex, which is subsequently trafficked to COP-I vesicles and LC3 autophagosomes. Then STING triggered the accumulation of LC3B puncta. The GPX4-STING complex subsequently fused with lysosomes and was encapsulated by autolysosome membrane marked by lysosomal-associated membrane protein 2B,^24^ ultimately leading to GPX4 degradation. These discoveries offer perspectives on the intricate mechanisms governing the degradation of GPX4 through autophagy, mediated by the interaction between STING and LC3B. In the absence of STING, the autophagic flux is impeded, resulting in the inability of GPX4 to colocalize with autophagosomes. Consequently, this disruption impedes the degradation of GPX4, which in turn mitigates cellular oxidative stress and ferroptosis. This enhanced understanding of the molecular mechanisms underlying GPX4-STING-autophagy interactions holds promise for the formulation of targeted treatment strategies applicable to related disease scenarios.

Given that the mechanisms governing ferroptosis are complex and remain incompletely comprehended, in an effort to decipher these intricacies, our research discloses a novel ferroptosis-regulatory mechanism. In this mechanism, STING is pinpointed as a partner protein that promotes the degradation of GPX4 during I/R injury. Unlike direct ubiquitination, STING’s role in GPX4 degradation likely involves its ability to regulate autophagy.^13^ Although STING is not an E3 ligase, it modulates autophagy by trafficking from the ER towards the ERGIC and further to the Golgi, promoting LC3 lipidation and autophagosome biogenesis. Our findings demonstrate that STING interacts with GPX4, facilitating its recruitment to autophagosomes for lysosomal degradation through the autophagic machinery rather than direct ubiquitination. Our study clarified the mechanism and highlighted STING-mediated autophagy’s broader implications in cell death pathways. Future investigations will focus on identifying potential E3 ligases or autophagy adaptors involved in this process to further elucidate the molecular mechanisms.

Our study not only deciphers the functional significance of STING in promoting myocardial ferroptosis during MI/R, but also delves into its protective effects on cardiac function subsequent to I/R injury. Notably, the investigations employing AAV-mediated GPX4 therapy further revealed that GPX4 functions as a downstream factor of STING. STING inhibitor H-151^43^ possesses the capacity to hinder the co-localization of STING and GPX4, disrupting autophagic flux, thereby diminishing ROS accumulation, and ultimately mitigating the severity of I/R injury. Together, the two effective treatment options demonstrate promising therapeutic potential in managing I/R injury, highlighting the substantial role of STING in regulating the degradation of GPX4 and the consequent myocardial ferroptosis during MI/R injury.

Mitochondria are pivotal determinants of myocardial cell survival or death during I/R.^44^ Specifically, our findings on mitochondrial function, health, and ATP status collectively demonstrate that blocking cGAS/STING signaling or activating GPX4 can limit disease progression and partially restore mitochondrial function. However, these approaches still result in defective mitochondria and impaired ATP production, highlighting the need for additional therapeutic strategies specifically targeting mitochondrial health to achieve complete cardiac repair.

Above all, by identifying STING as a prospective therapeutic target in the context of MI/R injury, we present a novel direction for future investigations into treatments for I/R. The formulation of a therapeutic approach aimed at augmenting GPX4 function or expression has the potential to mitigate the harmful impacts induced by STING activation during I/R injury on heart function. Translating cardiac protection from robust experimental evidence to clinical benefits for patients with acute myocardial infarction or undergoing cardiovascular surgery remains an urgent priority. To verify the clinical applicability of our animal findings, we conducted a study on a cohort of patients undergoing PCI treatment and confirmed the clinical relevance of our research, demonstrating the activation of the STING-GPX4 signaling axis in patients with ischemic heart disease, suggesting that it could potentially have a significant impact on I/R injury. Of course, a critical disparity between experimental and clinical studies lies in the presence of multiple co-morbidities and polypharmacy in patients, which are often inadequately modeled in animal studies. For instance, platelet inhibitors may limit the protective effects of experimental therapies for myocardial infarction, while propofol anesthesia can negate the benefits of ischemic conditioning. These factors highlight the importance of developing clinically relevant preclinical models to better align experimental therapies with the complexities of human disease.^45,46^

In conclusion, for the first time, our study unveiled the mechanisms through which STING initiates ferroptosis in CMs and exacerbates MI/R injury. We put forward a model in which MI/R stimulates the accumulation of cytoplasmic dsDNA, thereby activating cGAS-cGAMP-STING signaling. The activated STING then directly binds to GPX4 and induces autophagic degradation of GPX4, induces oxidative stress and eventually triggers ferroptosis in CMs. Specifically targeting STING might alleviate I/R-induced ferroptosis and cardiac damage. These results indicate that the STING-GPX4 axis plays a crucial role in myocardial ferroptosis. This not only discloses the novel molecular mechanisms underpinning GPX4-related cell death, but also designates STING as a promising therapeutic target for the management of MI/R injury.

## Materials and methods

### Ethics statements

All animal experiments were carried out in strict compliance with the National Institutes of Health (NIH) Guide for the Care and Use of Laboratory Animals. The Laboratory Animal Committee of Shandong University Qilu Hospital (No. DWLL-2021-206) gave its comprehensive approval, guaranteeing ethical and accountable execution during the entire research process. The clinical study was performed in line with the principles set forth in the Declaration of Helsinki and obtained approval from the Ethics Committee of Qilu Hospital, Shandong University (Approval Number: KYLL-2022(ZM)-1344). Informed consent was obtained from all patients who were enrolled in the study.

### Animal model of ischemia/ reperfusion (I/R)

For establishing the I/R animal model, male *C57BL/6J* mice aged 8 weeks were chosen. These animals were securely fixed on a mouse plate maintained at a constant temperature of 37 °C. Prior to the operation, the precordial area was thoroughly sterilized using iodophor. The mice were anesthetized using inhalation anesthesia with 3% isoflurane at a rate of 1 L/min. Using sterile scissors, a surgical incision of approximately 2 cm was made on the chest at the point where the heartbeat was most prominent. A 4-0 purse-string suture was placed, and the thorax above the heart was dissected through the fourth intercostal space using curved forceps. The chest was gently pressed to ease the heart out of the chest cavity and position it within the sutured opening, ensuring minimal air remained in the chest cavity. A 6-0 surgical suture was then used to tie a surgical slipknot around the left anterior descending coronary artery, with one end of the suture protruding outside the chest. Promptly, the heart was returned to the chest cavity, any excess air was expelled, and the epidermal incision was sutured closed. Post-operatively, the mice were transferred to the anesthesia recovery room. 45 min later, the slipknot for cardiac reperfusion was released, and the mice were returned to their rearing cages for continued care.^47^

Subsequently, the hearts were excised and stained to accurately determine the extent of myocardial necrosis, expressed as a percentage of the ischemic area at risk (AAR) that was not perfused. The ischemic region containing viable tissue was distinctly stained red using 2,3,5-triphenyltetrazolium, while the non-ischemic region was clearly marked blue with Evan’s blue. The hearts were then frozen at −80 °C for 10 min and cut into slices (5-6 slices/heart). Infarct, AAR, and LV areas were measured using ImageJ software from NIH. Infarct size was calculated as the ratio of infarct area to AAR area. These slices were photographed using a gross imaging microscope (Leica M205 FA), and the images were subsequently analyzed with the aid of NIH Image software.^48^

### Echocardiogram

Following a 24-h modeling phase, the cardiac structure and function of mice were evaluated using a Transthoracic Echocardiogram. The VisualSonic VeVo 2100 Imaging System, which originated from Toronto, Canada, was utilized for this assessment. Before commencing the procedure, the mice were anesthetized by inhaling 2% isoflurane. Subsequently, they were positioned on a heated platform kept at a temperature of 37 ± 1 °C and were securely attached to an electrocardiogram (ECG) for continuous monitoring. With the application of M-mode echocardiography, the left ventricular diastolic diameter (LVIDd) and left ventricular systolic diameter (LVIDs) on the parasternal long axis were carefully documented. Ultimately, the left ventricular ejection fraction and shortening fraction were computed automatically to enable a precise evaluation.

### Distinct cardiac cell populations isolation

The isolation of distinct cardiac cell populations from the infarcted hearts of mice was performed 24 h following myocardial ischemia-reperfusion (MI/R). This was done in accordance with pre-existing protocols.^49^ Standardized techniques were employed to isolate cardiomyocytes (CMs).^50^ For the isolation of cardiac fibroblasts (CFs) and macrophages, the Skeletal Muscle Dissociation Kit (Miltenyi Biotech, Shanghai, China) was utilized. To separate macrophages from CFs, Anti-F4/80-coated magnetic beads (Cat. No. 130-110-443; Miltenyi Biotech) were employed in strict accordance with the manufacturer’s instructions. Subsequently, the purified cells were gathered through centrifugation at 300 × *g* for 5 min at 4 °C, in preparation for the subsequent extraction of proteins.

### Immunofluorescence, Masson’s trichrome (MT), and 4-HNE staining

The infarct area was demarcated as the portion situated between the suture and the heart’s apex. The infarct border area was carefully characterized as the marginal zone that differentiates the infarcted tissue from the adjacent non-infarcted myocardium in the short-axis cross-section. Employing a microtome (RM2235; Leica Microsystems, Inc., Mannheim, Germany), heart tissues positioned 1 mm below the ligation site were accurately cut into 4-μm thick transverse slices, arranged along the horizontal long axis. Serial sectioning was applied for both MT staining processes, facilitating the assessment of myocardial fibrosis.

For the immunofluorescence staining of dsDNA, cGAS, STING, GPX4, and 4-HNE, the sections were first subjected to dewaxing. Antigen retrieval was then carried out using a dedicated kit (C1034; Solarbio, Beijing, China). Subsequently, the sections were immersed in 0.1% Triton X-100 (GC204003; Servicebio, Wuhan, China) in phosphate-buffered saline (PBS) for 10 min to increase their permeability. Next, they were incubated with 5% normal goat serum (G1208; Servicebio, Wuhan, China) in PBS for 60 min at room temperature (23–27 °C) to block non-specific binding sites. Antibodies specific to dsDNA, cGAS, STING, GPX4, or 4-HNE were then applied, and the sections were incubated overnight at 4 °C. After that, the sections were washed three times with PBS to eliminate unbound antibodies. Subsequently, incubation with Alexa Fluor 594 (ab150120; Abcam, Cambridge, MA, USA) and Alexa Fluor 488 (ab150081; Abcam, USA) secondary antibodies (diluted 1:200) was performed for 1 h in the dark at 37 °C to visualize the specific binding of the primary antibodies. Finally, the nuclei were counterstained with 4′,6-diamidino-2-phenylindole (DAPI, ab104139; Abcam, UK) to enable clear identification of cellular localization.

### Cell culture

HEK293T, HeLa, and HL-1 cells were procured from KeyGene BioTech in China. Mouse primary cardiomyocytes (MPCs) were isolated from *cgas*^*−/−*^, *Sting*^*−/−*^, *cgas*-CKO, *Sting*-CKO, as well as their control littermates or C57BL/6J wild-type (WT) mice. These cells were cultured in Dulbecco’s Modified Eagle Medium (DMEM) containing 10% fetal bovine serum (FBS; 9014-81-7, Sigma Aldrich, Germany). The cell cultures were maintained at a temperature of 37 °C.

### Cell IF staining and laser confocal analysis

In the process of immunocytochemistry staining, HeLa cells, HL-1 cells, or CMs were plated at a density of 3 × 10^5^ cells per milliliter on a 14-millimeter coverslip (WHB, China). Following treatment with ligands, the cells were immediately fixed using 4% paraformaldehyde (Beyotime, China) for 5 min. Next, cell membrane permeability was increased by treating the cells with 0.1% Triton X-100 (Beyotime, China) for an additional 5 min. Subsequently, non-specific binding sites were blocked by incubating the cells in a buffer containing 10% Donkey Serum (Solarbio Science & Technology, China) at room temperature for 1 h. Then, the samples were incubated with the specific primary antibodies overnight at 4 °C. After that, the samples were rinsed three times with PBS (Solarbio Science & Technology, China). The samples were then incubated with the assigned fluorescent secondary antibody for 1 h at room temperature, followed by another three rounds of rinsing with PBS. Ultimately, the samples were subjected to counterstaining with DAPI (ab104139; Abcam, UK) to mark the nuclei.

### Statistics analysis

Data were presented as the mean ± the standard error of the mean (SEM). All statistical analyses were carefully carried out with the use of GraphPad Prism 9 (GraphPad, San Diego, CA, USA). The SEM was denoted by the error bars. Initially, the normal distribution was examined, and subsequently, the Shapiro–Wilk test was applied for the assessment of variance homogeneity. The data exhibited approximate normal distribution (*P* > 0.05), indicating the suitability of parametric statistical methods. To assess statistically significant differences between two groups for normally distributed data, an unpaired two-tailed Student’s *t* test was employed. For non-normally distributed data, a nonparametric statistical method was employed. Specifically, the Kruskal–Wallis test was utilized, and this was followed by Dunn’s post-hoc test for conducting multiple comparisons. Differences were deemed statistically significant based on stringent criteria, specifically, *P < 0.05 for marginal significance, **P < 0.01 for moderate significance, ***P < 0.001 for strong significance, and ****P < 0.0001 for highly significant differences, or as not significant. At least three independent experiments was repeated to perform statistical analysis. Each data point included at least three biological repeats.

## Supplementary information

Supplymental materials

RAW Doubtful Echocardiography

RAW WB

## Data Availability

All data and materials be made available within the submitted material or in a public repository. The RNA-sequencing data generated in this study are publicly available in Gene Expression Omnibus (GEO) dataset GSE291453.
