# Supplementary material for: STING aggravates ferroptosis-dependent myocardial ischemia-reperfusion injury by targeting GPX4 for autophagic degradation
Source: Signal Transduct Target Ther. 2025 Apr 25;10:136. doi: 10.1038/s41392-025-02216-9 (PMC12022026; doi:10.1038/s41392-025-02216-9)
Supplement: Supplementary file 1 — Supplymental materials [file 41392_2025_2216_MOESM1_ESM.docx]

Supplementary Materials for

STING aggravates ferroptosis-dependent myocardial ischemia-reperfusion injury by targeting GPX4 for autophagic degradation

Xiaohong Wang, Tao Chen, Sizhe Chen, Jie Zhang, Liangyu Cai, Changhao Liu, Yujie Zhang, Xiao Wu, Na Li, Zhiyong Ma, Lei Cao, Qian Li, Chenghu Guo, Qiming Deng, Wenqian Qi, Yonghao Hou, Ruiqing Ren, Wenhai Sui, Haonan Zheng, Yun Zhang, Meng Zhang*, Cheng Zhang*

Correspondence to: [zhangmeng@sdu.edu.cn](mailto:xxxxx@xxxx.xxx)

**This PDF file includes:**

Materials and Methods

Figures S1 to S5

Tables S1 to S3

**Other Supplementary Materials for this manuscript include the following:**

None.

Materials and Methods

Materials

3×flag peptide (F4799) is purchased from Sigma Aldrich.

Mito Tracker™ Red CMXRos (M7512) were purchased from ThermoFisher Scientific.

BODIPY 493/503 (D3922), Lipofectamine Lip3000, Lipofectamine Lip2000 were purchased from Invitrogen.

Protein A/G agarose was purchased from Santa Cruz Biotechnology (USA).

HEPES (1939945) was purchased from Gibco.

MDA Assay Kit(ab118970), Cellular ROS Assay Kit(ab113851) and Mounting Medium with DAPI (ab104139) were purchased from Abcam.

KOD -Plus- Mutagenesis Kit (SMK-101) was purchased from Toyobo.

PrimeScript RT reagent Kit with gDNA Eraser (RR047A) was purchased from Takara.

Silver stain plus kit (P0017S) were purchased from Beyotime.

ELISA Kits for Transmembrane Protein 173 (MD 135452) and Glutathione Peroxidase 4 (MD 126635) was purchased from Medical Discovery Leader, China.

The antibodies were listed in Supplementary Table S1.

Agonists and inhibitors were listed in Supplementary Table S2.

Methods

*Animals:*  *cgas* and *Sting* general deficient mice on the *C57BL/6J* background (*cgas^-/-^* or *Sting^-/-^*) were obtained from Jackson Laboratory. Cardiomyocyte (CM) specific *cgas* knockout mice (*cgas*-CKO) were developed by Nanjing GemPharmatech Co., Ltd. through the clustered regularly interspaced palindromic repeats (CRISPR)/Cas9 system. Cardiomyocyte (CM) specific *cgas* knockout mice (*cgas*-CKO) were developed by Nanjing GemPharmatech Co., Ltd. through the clustered regularly interspaced palindromic repeats (CRISPR)/Cas9 system. Their genotyping was performed using the following primers: forward, 5′CCTTGTCTTAGGCAGCATGGAC3′ and reverse, 5′TTGACAGATGGTCCTCAGGCAC3′. The mice were crossed with *Myh6*-cre mice (*C57BL/6J*Gpt-H11em1Cin (*Myh6*-iCre)/Gpt, GemPharmatech Co., Ltd.#T004713) to obtain *cgas* ^fl/fl^; *Myh6*-cre mice. *Sting* CM-deficient mice (*Sting*-CKO) were also developed by Nanjing GemPharmatech Co., Ltd. using the CRISPR/Cas9 system, and their genotyping was performed using the following primers: forward, 5′TCCCAGGGTGAGCTTTGTAAAG3′ and reverse, 5′GGAGTTGGATCATGCTCACTGATC3′. The mice were crossed with *Myh6*-cre mice (*C57BL/6J*Gpt-H11em1Cin (*Myh6*-iCre)/Gpt, GemPharmatech Co., Ltd.#T004713) to obtain *Sting* ^fl/fl^; *Myh6*-cre mice. These mice were then backcrossed onto the *C57BL/6J* background (GemPharmatech Co., Ltd #N000013). To generate an ischemia/ reperfusion (I/R) animal model, eight-week-old male *cgas*-CKO, *Sting*-CKO, *cgas^-/-^*, *Sting^-/-^*, *C57BL/6J* and their control mice were used in the present study. Eight-week-old male *C57BL/6J* mice were purchased from GemPharmatech Co., Ltd. (Nanjing, China). All of the mice exhibited no difference in phenotypes. To mitigate aging's subcellular and molecular effects on myocardial cells and their cardioprotection against ischemia-reperfusion injury, we modeled with 8-week-old male mice,^1^ disregarding negligible gender differences.^2,3^ Yet, our mouse modeling has limitations. Although mice are widely used for studying molecular mechanisms, future studies must validate key findings in larger animal models or human systems to enhance translational relevance.^4^ Drawing from our preliminary experimental findings, we established statistical significance (α) at 0.05 and power (1-β) at 80% to ascertain the appropriate sample size. Consequently, five-six animals were meticulously analyzed within each group. These animals were randomly allocated into distinct groups in a blinded fashion to ensure unbiased results. All mice were housed in a sterile, pathogen-free animal facility to safeguard their welfare and maintain experimental integrity.

*Laser confocal detection:* The TUNEL, ROS, GFP-RFP-LC3, JC-1 and Mito-Tracker can be directly detected or incubated with Hochest (C1025; Beyotime, China) after staining. CD68, FSP1, Troponin, cGAS, STING, dsDNA, GPX4, 4-HNE, LC3B, LAMP2B, Flag and myc were labelled with primary antibodies and stained with fluorescent secondary antibody (Supplementary Table S1: Antibodies and manufacturers). Nuclei were stained with 4′,6-diamidino-2-phenylindole (DAPI) (ab104139; Abcam, UK) for 4 min. The heart tissues or several types of cells were subjected to microscopy (Zeiss LSM 900 confocal laser microscope or Zeiss Elyra7 with Lattice SIM [Oberkochen, Germany]). Representative images were randomly selected from each experimental group to ensure a comprehensive and unbiased representation of the staining patterns.

*IF assay for detecting cytoplasmic DNA:* CMs following anoxia/reperfusion were incubated with MitoTracker Red CMXRos (ThermoFisher Scientific, MA, USA) at 37°C for 30 min. Fixed and permeated into cells with BD Cytofix/Cytoperm solution (Becton, Dickinson and Company, NJ, USA). Subsequently, using 10% donkey serum to blocking nonspecific binding sites. Cells were incubated with anti-dsDNA antibodies (1:1000; Abcam, UK). After fluorescence secondary antibody labelling, the slides were observed using LSM900 Airyscan2 confocal laser microscope (Oberkochen, Germany).

*Live cell imaging:* Primary myocardial cells were pre-incubated with Fer-1^5^ for a duration of 12 h to effectively block the accumulation of reactive oxygen species (ROS). Subsequently, ROS live cell fluorescent probes (ab113851; Abcam, UK) were introduced to initiate the process of live cell recording. Determine the state of cells and weak expression of ROS. At the designated time points of 30.36 seconds, 2'3'-cGAMP (HY-12512; MCE, USA) was added to activate STING. The recording was terminated once the ROS levels stabilized, ensuring the capture of all relevant changes. Throughout this entire process, the cells were maintained in a sterile, normoxic environment at a constant temperature of 37 degrees. The Zeiss LSM900 confocal laser microscope (Oberkochen, Germany) was utilized to record the entire procedure, then subsequent analysis the imaging.

*Mouse primary cardiomyocyte (MPC) isolation and treatment:* Suckling mice were disinfected with 75% ethanol, and their chest cavities were surgically opened using ophthalmic scissors. The hearts were gently extracted and promptly excised with curved forceps, placed in a petri dish (539505; SARSTEDT, Germany) containing chilled DMEM/F12 (EH80023; eLGbio, China) medium. The atrial appendages were excised, residual blood gently removed, and the hearts transferred to fresh culture dishes with cold DMEM/F12. The hearts were uniformly dissected into ~1mm fragments and transferred to sterile centrifuge tubes. Digestive fluid, containing 0.125% pancreatic enzyme (25200-056; Gibco, USA) and 0.125% collagenase II(9001-12-1; Sigma Aldrich, Germany), was added at ~10x the tissue volume and incubated in a 37°C water bath with intermittent shaking to maintain suspension. Following an initial 10-minute digestion, the supernatant was discarded following natural sedimentation. Digestion resumed with fresh enzyme (~10x sediment volume) for 15 min, with intermittent shaking. The supernatant was then transferred to a new tube, and digestion terminated by adding culture medium with 20% serum([9014-81-7](https://www.sigmaaldrich.cn/CN/zh/search/9014-81-7?focus=products&page=1&perpage=30&sort=relevance&term=9014-81-7&type=cas_number), Sigma Aldrich, Germany). Remaining myocardial fragments were further digested until complete. The combined supernatants were centrifuged at 400×g for 8 min, discard the supernatant, and the cells gently resuspended in appropriate culture medium. The cell suspension is filtered through a 40 μm cell strainer (BS-40-XBS; biosharp, China) to eliminate cell clumps. Subsequently, cells are plated in a 100 mm dish for differential adhesion lasting 90 min to eliminate non-myocardial cells, including myocardial fibroblasts and vascular cells. The supernatant is aspirated gently, and the remaining cells are filtered through a 40 μm filter. Bromodeoxyuridine (BrdU) is added to the culture medium to attain a final concentration of 0.1 mmol/L. After gentle mixing, the cells are seeded in a gelatin-coated 6-well plate. Following incubation in a 5% CO_2_ atmosphere at 37°C for 48 h, the cells are washed once with serum-free DMEM/F12. Complete culture medium containing 0.1 mmol/L BrdU is added and replaced every 24 h. Prior to seeding, 10 μl cell suspension is mixed with 10 μL trypan blue (C0040; solarbio, China) staining solution for 3 min to assess cell viability using a cell counter (ThermoFisher Scientific, MA, USA).

*Adeno-associated virus (AAV) delivery:*

Cell: mRFP-GFP-LC3 autophagy double labeled AAVs was purchased from Hanbio (Shanghai, China). When infected with the virus, add 1/2 volume of fresh culture medium, and after 4 h of AAVs infection (MOI:100), replenish the volume to the original level.

Animal: An AAV 9 system was used to deliver GPX4 and its vector to the corresponding treated mice. The serotype 9 AAVs specifically encoding *c*-*TNT* *gpx4* (Mus-*gpx4*-c*TNT*-*C*-GFP) were designed by BioSune Biotechnology (Shanghai, China). Every mouse was administered 5×10^11^ vg AAV9, which carried the GPX4 gene or negative controls, through the tail vein for 14 days. After that, the transfection efficiency of AAVs was verified.

*Plasmid transfection:* Myc-tagged STING (Myc-STING) was described before.^6^ The complementary DNA (cDNA) of GPX4 was amplified from both human and mouse cells using standard polymerase chain reaction (PCR) and cloned into pCMV2. Flag-tagged GPX4 (Flag-GPX4) was constructed by BioSune Biotechnology (Shanghai, China). Additionally, the truncated mutant was constructed using a KOD Plus mutagenesis kit (TOYOBO Life Science, JAPAN) based on wide-type plasmids. Transfect HeLa, HEK293T and HL-1 cells using lipofectamine 3000 (Invitrogen, MA, USA) reagent. hGPX4 cDNA was subcloned into the pCMV-Flag plasmid, and the Flag-hGPX4 plasmid was used as template to construct Flag-GPX4(△*G*126), Flag-GPX4(△*R*127), and Flag-GPX4(△*N*146). Using Myc-STING plasmid as the template, Myc-STING (△*Y*167), Myc-STING (△*E*260), Myc-STING (△*Y*245), Myc-STING (△*Q*266) and Myc-STING (△*T*267) were obtained through PCR amplification. The mutant mutates the corresponding base to alanine (A). All constructs were confirmed via DNA sequencing.

*Western blot (WB) and analysis:* To extract total protein from cardiomyocytes, a protease inhibitor mixture (Sigma Aldrich, Germany) was used for cell lysis. The lysate was centrifuged at 4°C for 15 min at 12000×g to pellet cellular debris. Protein concentrations were adjusted using the bicinchoninic acid (BCA) assay (ThermoFisher Scientific, MA, USA) and samples were boiled at 95°C for denaturation. Sodium dodecyl sulphate-polyacrylamide gel electrophoresis (SDS-PAGE) was employed to separate the protein components of the cell lysate, and the resolved proteins were transferred onto a polyvinylidene fluoride (PVDF) membrane. Membrane blocking was achieved with 3% BSA at room temperature for 1 hour, followed by overnight incubation with a primary antibody specific for the target protein (Supplementary Table 1) at 4°C. After washing, a secondary antibody was applied, and the membrane was incubated at room temperature for 1 hour before extensive washing. Detection of protein signals was achieved through the addition of Immobilon® ECL Ultra Western HRP substrate (Millipore, CA, USA), and images were captured using a luminescent image analyser (Amersham Imager 600, GE, USA). Protein band intensities were quantified using Image J software (version 1.52a, NIH, USA), and the data were normalised to glyceraldehyde 3-phosphate dehydrogenase (GAPDH) levels. The results are presented as mean ± standard deviation (SD).

*Endogenous Co-immunoprecipitation and WB analyses:* MPCs were cultured following anaerobic reperfusion, with the optional addition of 2'3'-cGAMP. Subsequently, cells were harvested and lysed using RIPA buffer (Solarbio, China) containing a protease inhibitor mixture (Sigma Aldrich, Germany). Cell debris and insoluble proteins were removed by centrifugation at 12,000×g for 10 min. The supernatant was collected, and each sample (1 mg) were incubated overnight at 4°C with either anti-STING or anti-GPX4 antibodies, or non-specific rabbit IgG. Protein A/G PLUS Agarose (Santa Cruz Biotechnology, USA) was added, and incubation continued for 4 h at 4°C. The beads were washed five times with RIPA buffer, boiled in protein loading buffer to elute the immunoprecipitates, separated using 2× SDS-PAGE sample buffer, and analyzed by Western blot (WB) with anti-GPX4, anti-STING, or anti-GAPDH antibodies.

*Exogenous Co-immunoprecipitation and WB analyses:* HeLa cells were lysed with a cell lysis reagent (Sigma Aldrich, Germany) containing a cocktail of phosphatase (Roche, Switzerland) and protease inhibitors (Sigma Aldrich, Germany). Protein concentration was determined using BCA (ThermoFisher Scientific, USA). The lysate was resuspended in RIPA buffer (Solarbio, China) and centrifuged at 4°C for 15 min. The supernatant was collected, and a 60 μL aliquot was reserved as the 'Input' sample for subsequent WB analysis. The clarified lysate was incubated with Flag or Myc magnetic beads (Sigma Aldrich, Germany) on a rocker platform at 4°C for 6 h. The beads were washed five times with 1 mL Tris-buffered saline with Tween® 20(TBS-T; T8220; Solarbio, China) and eluted with competitive peptides, followed by boiling in protein loading buffer at 95°C for 10 min. The samples were resolved by SDS-PAGE and transferred to a PVDF membrane using a Bio-Rad vertical electrophoresis system. Immunoblotting was performed to assess the input and eluted immunoprecipitates from exogenous co-immunoprecipitation experiments.

*LC-MS/MS analysis of STING:* After treatment with ischemia-reperfusion (I/R), cardiomyocytes (CMs) were processed to extract total protein, and the supernatant was obtained through centrifugation. Subsequently, a specific affinity antibody targeting the STING protein (10 mg) was added to the supernatant for a 4-hour tandem affinity purification procedure. To verify the antibody's effectiveness, a portion of the immunopurified material was processed using a rapid silver staining kit (Beyotime, China). The remaining samples were then prepared for SDS-PAGE analysis. The bromophenol blue front was allowed to migrate to a length of 1 cm within the separation gel, followed by the removal of the concentrated gel. The separation gel was subsequently cut, and the gel was moistened with deionised water after staining with Coomassie brilliant blue (Epizyme, MA, USA) and subsequent decolorization. For the identification of STING cross-linked protein partners, LC-MS/MS analysis was performed on a Q-Exactive mass spectrometer (ThermoFisher Scientific, MA, USA), coupled with Easy nLC (ThermoFisher Scientific, MA, USA). This analysis ran for 120 min, providing comprehensive data on the interacting proteins with STING.

*Reference transcriptome experiment and bioinformatics analysis (RNA-sequencing):* RNA-seq was performed by LC-Bio Technologies (Hangzhou) CO., LTD. *Sting*-CKO or *Sting* ^fl/fl^ I/R model mice heart were randomly selected, and the border of the infarction area was cut and quickly rinsed with pre-cooled RNase-free water. To prevent RNA degradation, clean tissue was maintained on ice and promptly transferred into a frozen tube. Immediately following, the tissue was frozen using liquid nitrogen for a duration exceeding 15 min, and the samples were subsequently shipped on dry ice. Only after the samples met the necessary quality standards was the reference transcription RNA sequencing experiment conducted. The selection of differentially expressed mRNAs was based on a fold change greater than 2 or less than 0.5, coupled with a parametric F-test comparing nested linear models using the R package edgeR, with a significance threshold of P<0.05. Finally, enrichment analyses were conducted using the DAVID software, encompassing both Gene Ontology (GO) and Kyoto Encyclopedia of Genes and Genomes (KEGG) annotations.

*Mitochondrial Oxygen Consumption Rate Detection:* The oxygen consumption rate (OCR) was measured utilizing a XFe96/XF Pro plate (Seahorse, Agilent Cell Analysis Technology, USA). Cells were seeded at a density of 1.2 × 10^5^ per well and allowed to settle naturally for an hour in a sterile environment. Following this, the cell plate was incubated overnight to promote cell adhesion. Once the cells achieved 70–80% confluence, they were exposed to the respective stimuli and underwent the OGD/R model construction. Subsequently, 2 μm oligomycin, 1 μm FCCP, and 0.5 μm rotenone/antimycin A were sequentially administered. The OCR was then measured using an extracellular flux analyzer under mitochondrial stress test conditions.

*Blood Sample Collection and Cytokine:* A total of 15 patients with acute myocardial infarction (AMI) who underwent emergency PCI were enrolled after satisfying inclusion and exclusion criteria. Blood samples were collected from all patients and divided into two groups: pre-PCI and post-PCI. The concentrations of STING and GPX4 were measured using ELISA kits (Medical Discovery Leader, China). The baseline characteristics of the patients involved in the study are detailed in Supplementary Table S3.

*Human Embryonic Stem Cells Induced Cardiomyocytes:* Human cardiomyocytes prepared as per prev. protocol.^7^ H9 embryonic stem cells differentiated into cardiomyocytes using chem.-defined, xenofree, small mol.-based method. Cultured on 6-well plate precoated with Matrigel (BD Biosciences, catalog no. 354277) in E8 medium (basal medium, Life Technologies, catalog no. A1517001). At 70% confluence, medium replaced with 6 μmol/L CHIR99021 (Selleckchem, catalog no. S1263-25 mg). After 48 hours, changed to 2 μmol/L Wnt-C59 (Biorbyt, catalog no. orb181132) for another 48 hrs. Maintained in basal medium, changed every 48 hours. Beating cardiomyocytes appeared ~day 8. On day 10, replaced with glucose-free RPMI 1640 (Life Technologies, catalog no. 11879020) for purification. Two days later, dissociated using TrypLE (Life Technologies) for 10 mins and passaged to Matrigel-precoated plate.

*Picture drawing:* Part of the image was drawn by *Figdraw*.

**
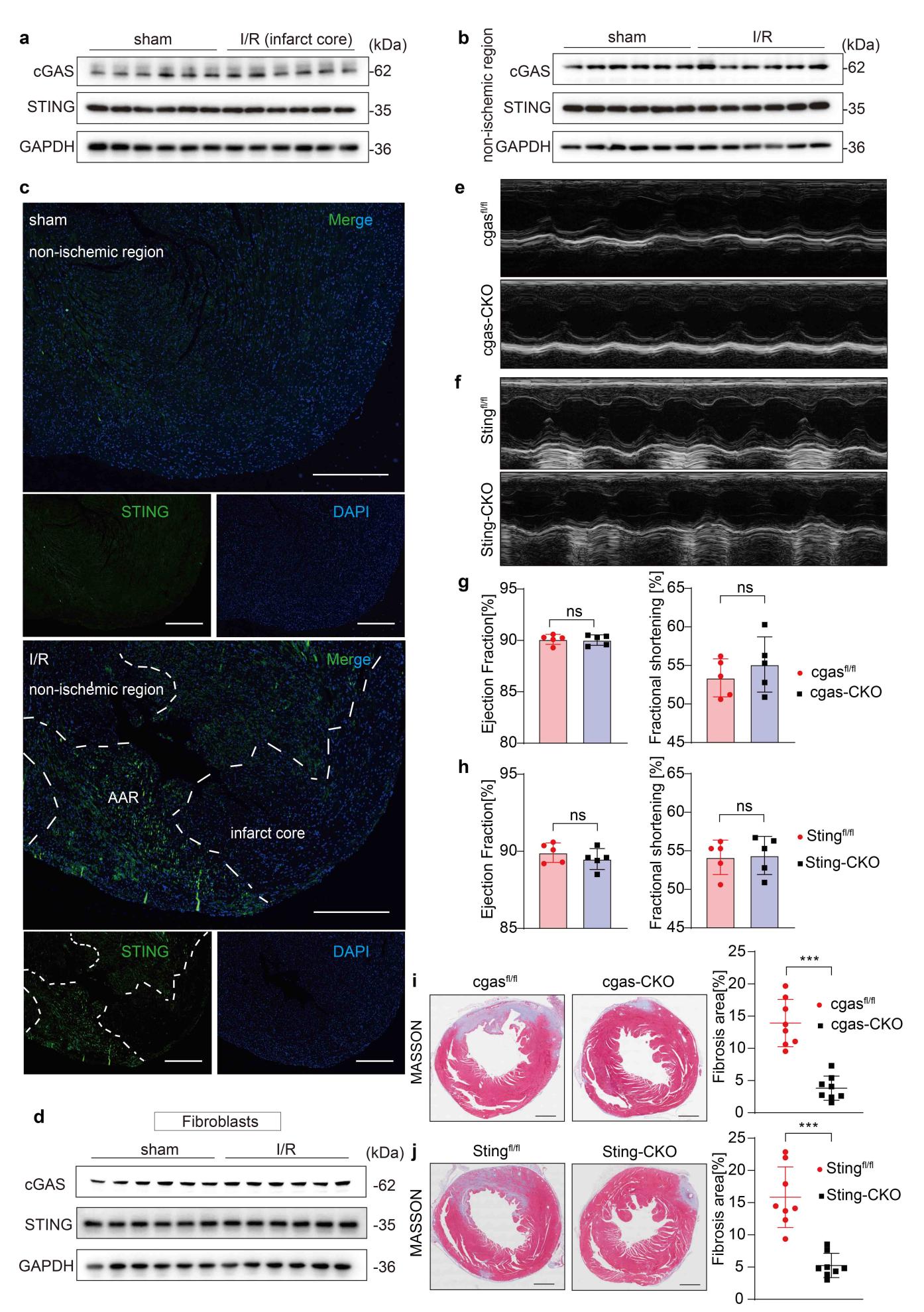
**

Supplementary Fig.1 | Expression of cGAS and STING in different cardiac regions and cell types, baseline cardiac function in knockout and WT mice, and fibrosis levels post-7-day-I/R.

**a,** Western blotting of cGAS and STING level in CMs isolated from infarct core post-I/R or sham. **b**, Western blotting of cGAS and STING level in CMs isolated from non-ischemic region post-I/R or sham. **c**, Immunofluorescence analysis for detecting STING in heart section post-I/R. The positive reaction for STING is displayed in green. Scale bar=500 μm. **d**, Western blotting of cGAS and STING level in fibroblasts isolated from heart post-I/R or sham. **e-h**, Baseline data of cardiac function in *cgas*^fl/fl^, *cgas*-CKO, *Sting*^fl/fl^ and *Sting*-CKO mice (n=5). **i**, Masson staining and measured fibrosis area% in *cgas*^fl/fl^ or *cgas*-CKO mice post-7-day-I/R (n = 8). Scale bar=1mm. **j**, Masson staining and measured fibrosis area% in *Sting*^fl/fl^ or *Sting*-CKO mice post-7-day-I/R (n = 8). Scale bar=1mm. Mean ± SEM. NS, non-significance, ****P* < 0.001; I/R, Ischemia reperfusion; cGAS, cyclic guanosine monophosphate-adenosine monophosphate synthase; STING, stimulator of interferon genes; CM, cardiomyocyte; *cgas*-CKO, *cgas*^fl/fl^ *Myh*^6iCre^; *Sting*-CKO, *Sting*^fl/fl^ *Myh*^6iCre^.

**
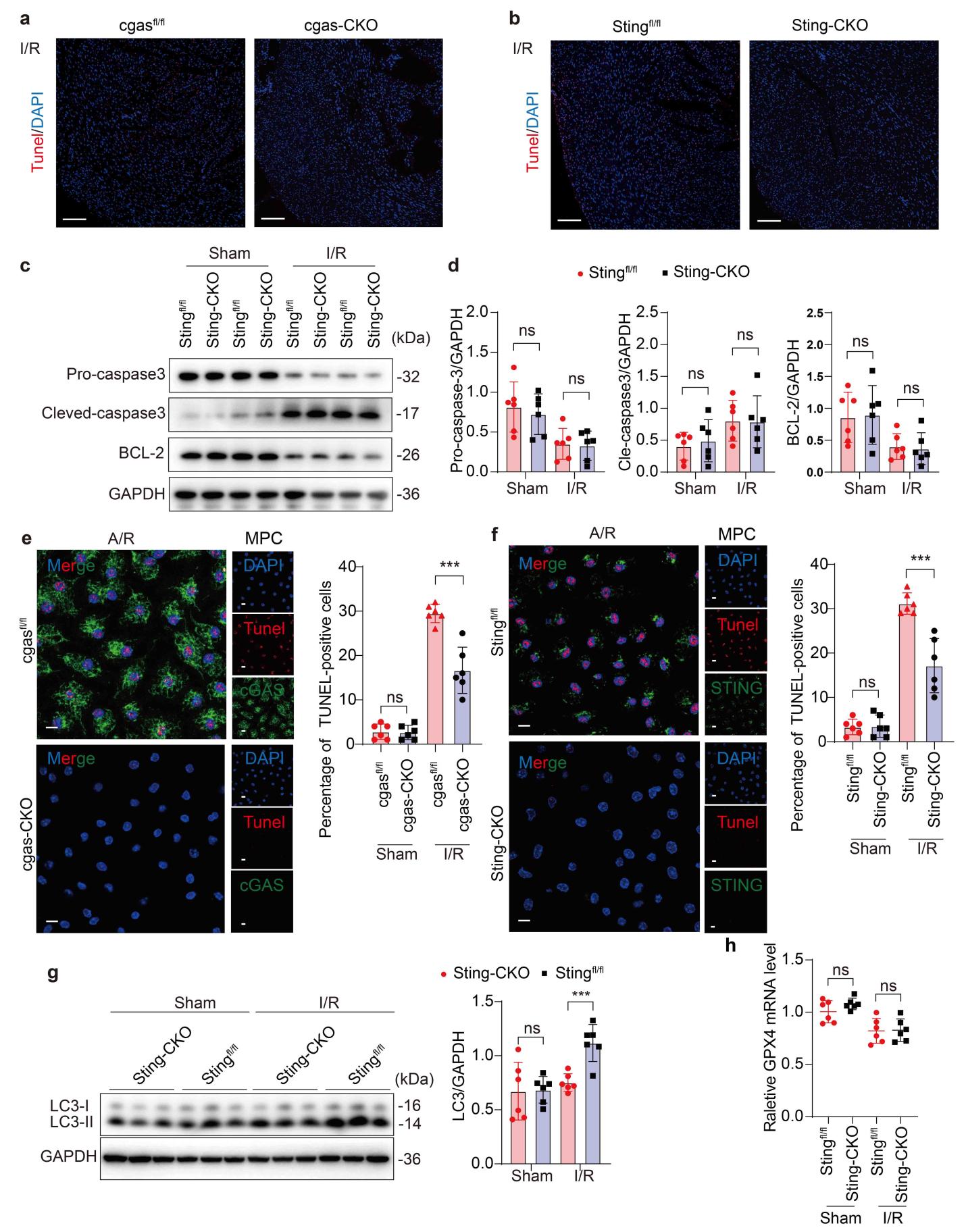
**

Supplementary Fig.2 | Effect of cGAS or STING knockout on I/R-induced cell death, apoptosis-related protein levels, and markers of oxidative stress in cardiomyocytes.

**a-b**, Effect of cGAS or STING knockout (CKO) on I/R-induced cell death was evaluated: (**a**), Immunofluorescence analysis for detecting TUNEL in heart section of the *cgas*^fl/fl^ or *cgas*-CKO border region post-I/R. Positive reaction for TUNEL is displayed in red and co-localization with DAPI. Scale bar=200 μm. (**b**), Immunofluorescence analysis for detecting TUNEL in heart section of the *Sting*^fl/fl^ or *Sting*-CKO border region post-I/R. Positive reaction for TUNEL is displayed in red and co-localization with DAPI. Scale bar=200 μm. **c**, Western blotting of Pro-caspase3, Cleved-caspase3 and BCL-2 in *Sting*^fl/fl^ or *Sting*-CKO CMs isolated from border region post-I/R or sham. **d**, Statistical chart of C (n = 6). **e-f**, Effect of cGAS or STING knockout (CKO) in CMs on A (95% nitrogen/5% CO_2_)/R (room air/5% CO_2_)-induced cell death was evaluated: (**e**), Double immunofluorescence analysis for detecting TUNEL and cGAS in *cgas*^fl/fl^ or *cgas*-CKO MPCs at least six times. Positive reaction for TUNEL is displayed in red and co-localization with DAPI. The positive reaction for cGAS is displayed in green. Scale bar=10 μm.

(**f**), Double immunofluorescence analysis for detecting TUNEL and STING in *Sting*^fl/fl^ or *Sting*-CKO MPCs at least six times. The positive reaction for TUNEL is displayed in red and co-localization with DAPI. The positive reaction for STING is displayed in green. Scale bar=10 μm. **g**, Western blotting of LC3-I and LC3-II in *Sting*^fl/fl^ or *Sting*-CKO CMs isolated from border region post-I/R or sham. **h,** Representative GPX4 mRNA levels in *Sting*^fl/fl^ or *Sting*-CKO MPCs post-I/R or sham, as observed via RT-PCR (n=6). Mean ± SEM. NS, non-significance, ***P < 0.001. Ctrl, control; BCL-2, B-cell lymphoma-2; TUNEL, Terminal Deoxynucleotidyl Transferase mediated dUTP Nick-End Labeling; A/R, anoxia/reoxygenation; MPC, mouse primary myocardial cells.

**
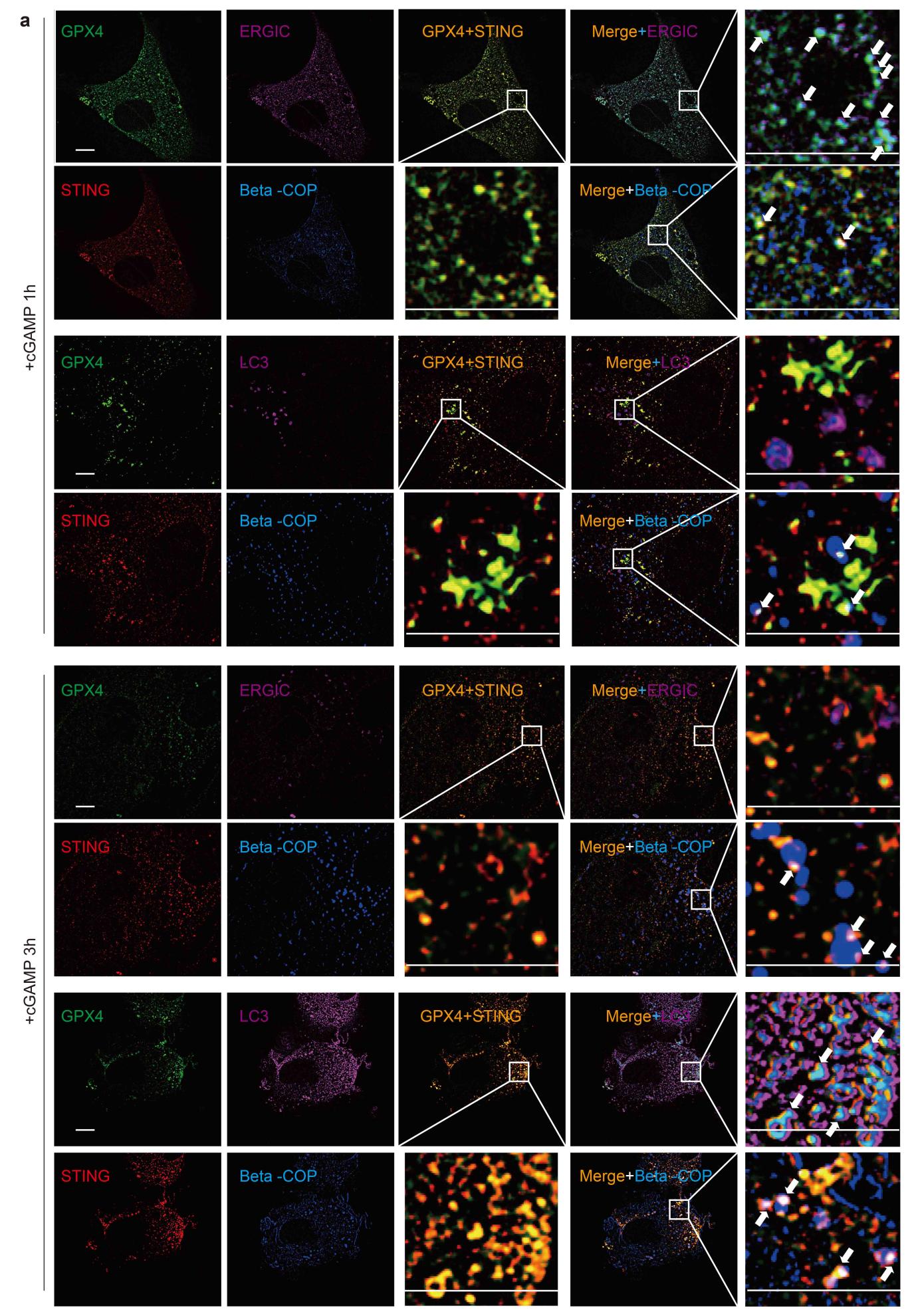
**

Supplementary Fig.3 | cGAMP induces trafficking of STING-GPX4 complex to the ERGIC, COP-I vesicles and LC3 autophagosomes.

**a**, MPCs were stimulated with cGAMP for the indicated time. Cells were immunostained with antibodies specific for GPX4, STING, ERGIC53 (ERGIC), beta-COP (COP-I vesicles) or LC3, followed by fluorescence microscopy. The positive reaction for GPX4 is displayed in green, the positive reaction for STING is displayed in red, the positive reaction for ERGIC or LC3 is displayed in purple, the positive reaction for COP-I is displayed in blue. The STING-GPX4 complex is yellow or orange (Merge) and turns light blue with ERGIC or LC3, white with COP-I. The arrow marks co-localization. Scale bar=5 μm. ERGIC, ER-Golgi intermediate compartment.

**
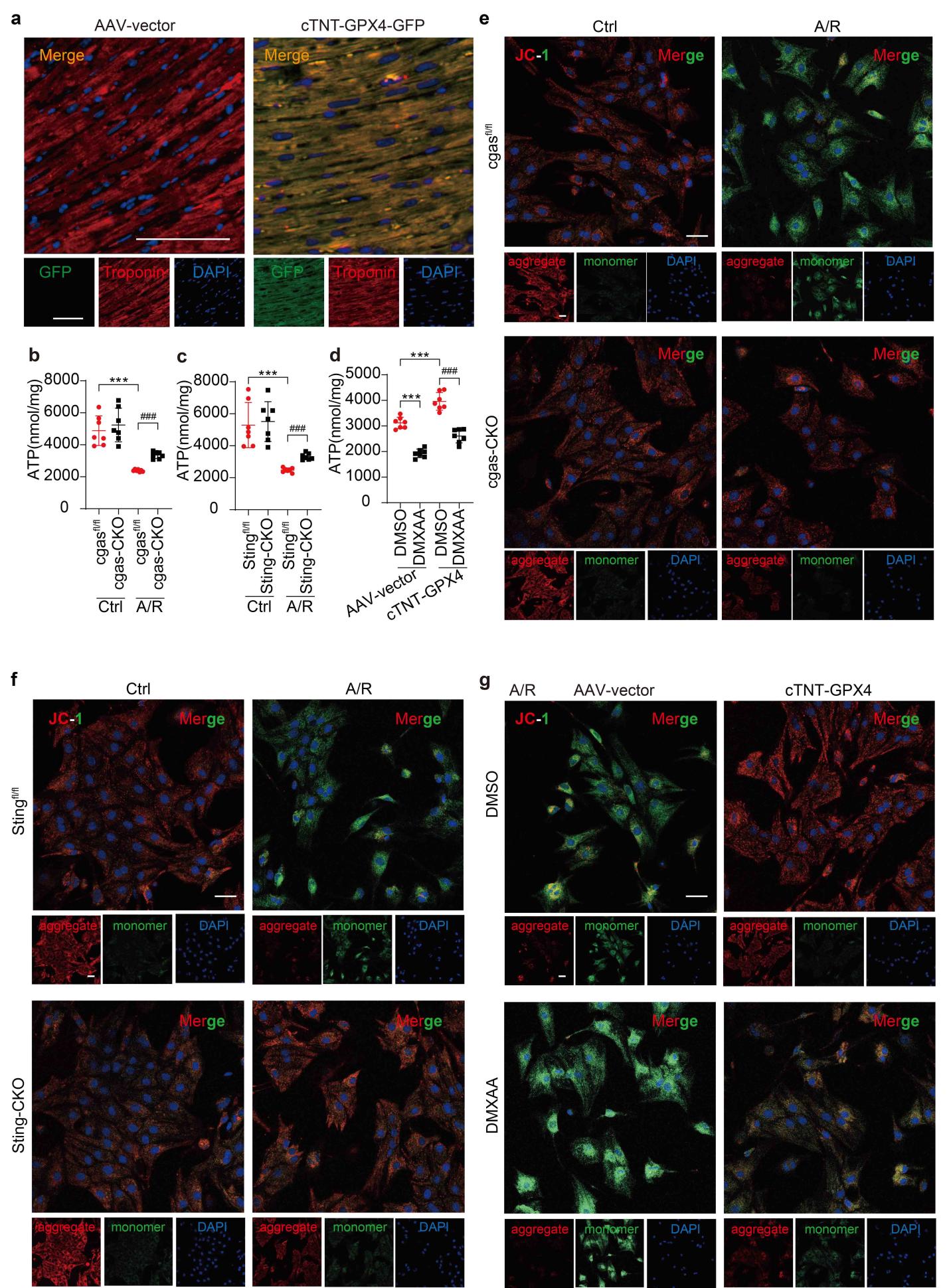
**

**Supplementary Fig.4** | **Verification of GPX4 overexpression in the heart tissue, and evaluation of ATP levels and mitochondrial membrane potential in cardiomyocytes with cGAS or STING knockout or GPX4-overexpressing phenotypes.**

**a**, Double immunofluorescence analysis for detecting markers of CMs and cTNT-GPX4-C-GFP in the same heart section post I/R or Sham to verify successful overexpression of GPX4 in CMs; the positive reactions of tissue sections are displayed in green (GFP) and red (Troponin). The positive reaction for co-localisation is displayed in yellow. Scale bar=200 μm. **b-d**, ATP level (n=7) in (**b**), *cgas*^fl/fl^ or *cgas*-CKO MPCs post-A/R or control (**c**), *Sting*^fl/fl^ or *Sting*-CKO MPCs post-A/R or control (**d**), and MPCs loading with cTNT-GPX4 or AAV-vector post-A/R, with or without DMXAA. **e-g**, The effect of mitochondrial membrane potential of (**e**), *cgas*^fl/fl^ or *cgas*-CKO MPCs post-A/R or control (**f**), *Sting*^fl/fl^ or *Sting*-CKO MPCs post-A/R or control (**g**), MPCs loading with cTNT-GPX4 or AAV-vector post-A/R, with or without DMXAA (n=5). Scale bar=20 μm. Mean ± SEM. ***P < 0.001 and ###P<0.001. AAV, adeno-associated virus; GPX4, glutathione peroxidase 4.

**
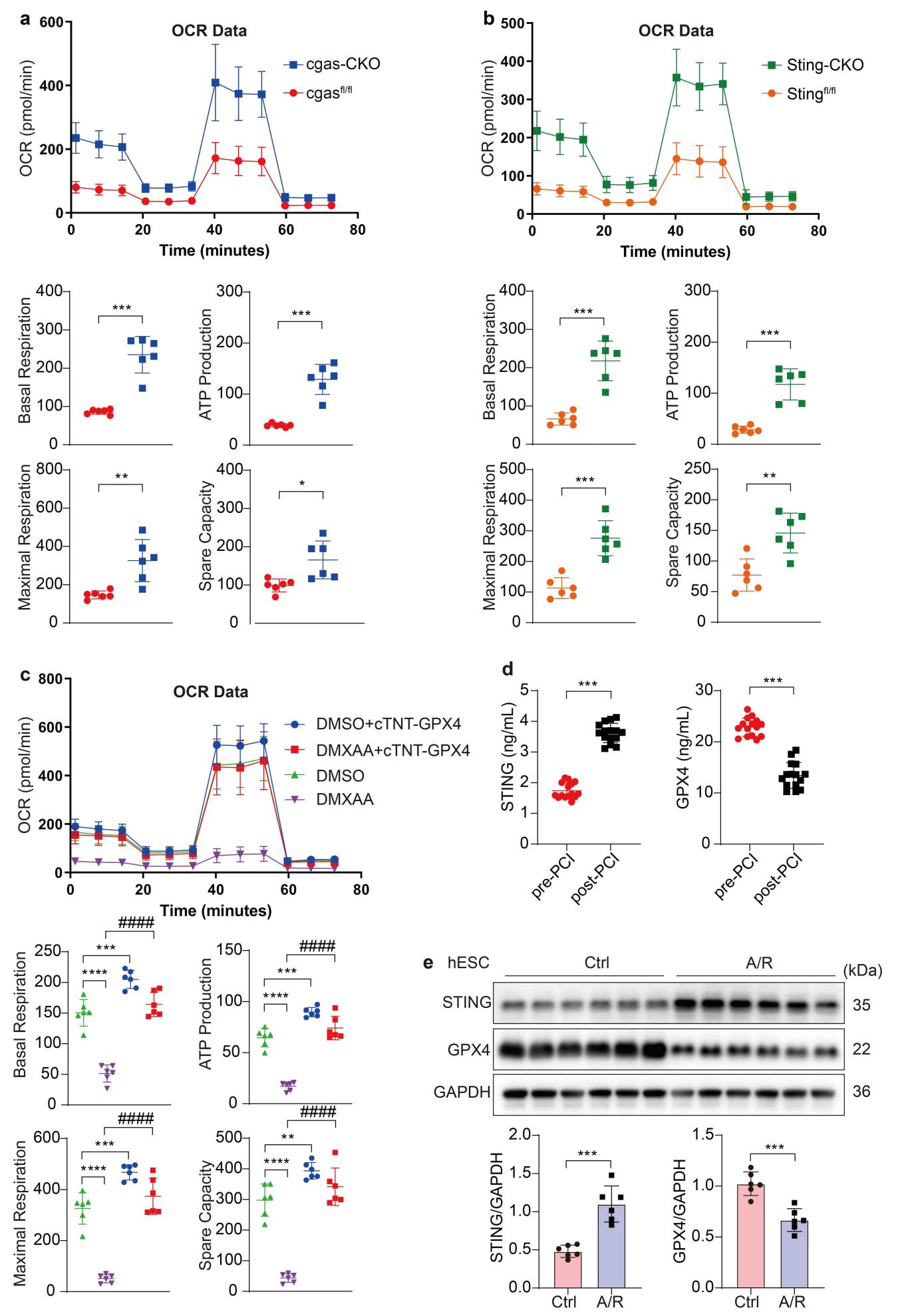
**

**Supplementary Fig.5** | **Mitochondrial stress tests in cGAS or STING knockout and GPX4-modified MPCs post-A/R, serum levels of STING and GPX4 in PCI patients, and protein expression in cultured human cardiomyocytes post-A/R**

**a-c**, Mitochondrial stress tests were conducted on MPCs post-A/R in (**a**), *cgas*^fl/fl^ or *cgas*-CKO mice (**b**), *Sting*^fl/fl^ or *Sting*-CKO mice (**c**), MPCs loading with cTNT-GPX4 or AAV-vector, with or without DMXAA (n=6). **d,** Serum levels of STING and GPX4 before and 12-h after PCI of patients (n=15). **e**, Representative Western blots and averaged data of STING or GPX4 from cultured human cardiomyocytes derived from human embryonic stem cells post-A/R or not (n=6). Mean ± SEM. NS, non-significance, *P < 0.05, **P < 0.01, ***P < 0.001, ****P < 0.0001 and ####P<0.0001; PCI, percutaneous coronary intervention; hESC, human embryonic stem cell.

Supplementary table 1: Antibodies and manufacturers

| Antibody | Company | Catalog No. | Concentrations |
| --- | --- | --- | --- |
| STING | Cell Signaling Technology | 13647S | Western blot: 1:1000,  IP: 1:100 |
| STING | RD | AF6516 | IF: 1:100 |
| GPX4 | Abcam |  | Western blot: 1:1000,  IF: 1:100 |
| GAPDH | Cell Signaling Technology | 2118 | Western blot: 1:1000 |
| Anti-dsDNA | Abcam | ab273137 | IF: 1:100 |
| P62 | Cell Signaling Technology | 23214 | Western blot: 1:1000 |
| LAMP2B | Abcam | ab118959 | IF: 1:200 |
| LC3B | Abcam | ab192890 | Western blot: 1:1000,  IF: 1:200 |
| FSP1 | Proteintech | No.16105-1-AP | IF: 1:200 |
| Troponin | Abcam | ab209809 | IF: 1:200 |
| Caspase-3 | Abcam | ab32351 | Western blot: 1:1000 |
| BCL-2 | Abcam | ab182858 | Western blot: 1:1000 |
| ACSL4 | Abcam | ab155282 | Western blot: 1:1000 |
| SLC7A11 | Cell Signaling Technology | 98051 | Western blot: 1:1000 |
| CD68 | Abcam | ab125212 | IF: 1:200 |
| Anti-4 Hydroxynonenal | Abcam | ab48506 | IF: 1:200 |
| Myc | Cell Signaling Technology | 2278 | Western blot: 1:1000 |
| Flag | Sigma-Aldrich | F1804 | Western blot: 1:1000 |
| His | Cell Signaling Technology | 12698S | Western blot: 1:1000 |
| HA | ORIGENE | TA180128 | Western blot: 1:1000 |
| IPKine mouse anti rabbit IgG light chain secondary antibody | Abbkine | A25022 | Western blot: 1:1000 |
| Goat anti mouse IgG H&L (Alexa Fluor® 488) Pre adsorption secondary antibody | Abcam | ab150177 | IF: 1:200 |
| Goat anti rabbit IgG H&L (Alexa Fluor® 488) Pre adsorption secondary antibody | Abcam | ab150081 | IF: 1:200 |
| Goat anti mouse IgG H&L (Alexa Fluor® 594) Pre adsorption secondary antibody | Abcam | ab150120 | IF: 1:200 |
| Goat anti rabbit IgG H&L (Alexa Fluor® 488) Pre adsorption secondary antibody | Abcam | ab150084 | IF: 1:200 |
| Goat anti mouse IgG H&L (Cy5) Pre adsorption secondary antibody | Abcam | ab6563 | IF: 1:200 |
| Goat anti rabbit IgG H&L (Cy5) Pre adsorption secondary antibody | Abcam | ab97077 | IF: 1:200 |
| Donkey anti rabbit IgG H&L (Alexa Fluor® 405) Pre adsorption secondary antibody | Abcam | ab175649 | IF: 1:200 |
| Donkey anti sheep IgG H&L (Alexa Fluor® 488) Pre adsorption secondary antibody | Abcam | ab150177 | IF: 1:200 |
| cGAS | Cell Signaling Technology | 31659 | IF: 1:200 |

Supplementary table 2: Agonists, inhibitors, and manufacturers

| Drug | Company | Catalog No. | Concentrations |
| --- | --- | --- | --- |
| 2’3’-cGAMP | MCE | HY-12512 | In vitro:3 µg/mL,2h |
| Fer-1 | MCE | HY-100579 | In vitro:5μM,12h |
| Erastin^5^ | MCE | HY-15763 | In vitro:10μM,12h |
| DMXAA | MCE | HY-10964 | In vitro:150µg/mL,2h  In vivo:  5mg/kg body weight,  i.p. daily for 3 days |
| Wortmannin | MCE | HY-10197 | In vitro:100nM,24h |
| 3-MA | MCE | HY-19312 | In vitro:100μM, 24h |
| LY294002 | TargetMil | T2008 | In vitro:75μM, 24h |
| NH4CL | Sigma-Aldrich | A9434 | In vitro:4mM, 12h |
| Bafilomycin A1 | MCE | HY-100558 | In vitro:50nM, 24h |
| chloroquine | MCE | HY-17589A | In vitro:20μM, 24h |
| calpeptin | MCE | HY-100223 | In vitro:100nM, 24h |
| MG-132 | MCE | HY-13259C | In vitro:10μM, 1h |

**Supplementary table 3**: Clinical characteristics of patients.

| Age (years) | 60.6±14.267 |
| --- | --- |
| Men (n, %) | 15 (100%) |
| Body mass index (kg/m^2^) | 25.862±4.404 |
| Smoking (n, %) | 9 (60%) |
| Hypertension (n,%) | 1 (6.67%) |
| Cho (mmol/l) | 4.073±1.074 |
| HDL-C (mmol/l) | 1.081±0.218 |
| LDL-C (mmol/l) | 2.612±0.957 |
| TG (mmol/l) | 1.206±0.635 |

Data were expressed as mean±SEM or number (n, %) of participants.

Cho, cholesterol; HDL-C, high-density lipoprotein cholesterol; LDL-C, low-density

lipoprotein cholesterol; TG, triglyceride.

**Reference**

1 Boengler, K., Schulz, R. & Heusch, G. Loss of cardioprotection with ageing. *Cardiovasc Res*. **83**, 247-261, (2009).

2 Kleinbongard, P., Lieder, H., Skyschally, A. & Heusch, G. No sex-related differences in infarct size, no-reflow, and protection by ischaemic pre-conditioning in Göttingen minipigs. *Cardiovasc Res*. **119**, 561-570, (2023).

3 Guo, Y. *et al.* Genetic background, gender, age, body temperature, and arterial blood pH have a major impact on myocardial infarct size in the mouse and need to be carefully measured and/or taken into account: results of a comprehensive analysis of determinants of infarct size in 1,074 mice. *Basic Res Cardiol*. **107**, 288, (2012).

4 Ferdinandy, P. *et al.* Interaction of Cardiovascular Nonmodifiable Risk Factors, Comorbidities and Comedications With Ischemia/Reperfusion Injury and Cardioprotection by Pharmacological Treatments and Ischemic Conditioning. *Pharmacol Rev*. **75**, 159-216, (2023).

5 Xie, Y. *et al.* Ferroptosis: process and function. *Cell Death Differ*. **23**, 369-379, (2016).

6 Liu, B. *et al.* The ubiquitin E3 ligase TRIM31 promotes aggregation and activation of the signaling adaptor MAVS through Lys63-linked polyubiquitination. *Nat Immunol*. **18**, 214-224, (2017).

7 Shan, D. *et al.* Cardiac Ischemic Preconditioning Promotes MG53 Secretion Through H(2)O(2)-Activated Protein Kinase C-δ Signaling. *Circulation*. **142**, 1077-1091, (2020).
