# Supplementary figures and images for: STING aggravates ferroptosis-dependent myocardial ischemia-reperfusion injury by targeting GPX4 for autophagic degradation
Source: Signal Transduct Target Ther. 2025 Apr 25;10:136. doi: 10.1038/s41392-025-02216-9 (PMC12022026; doi:10.1038/s41392-025-02216-9)

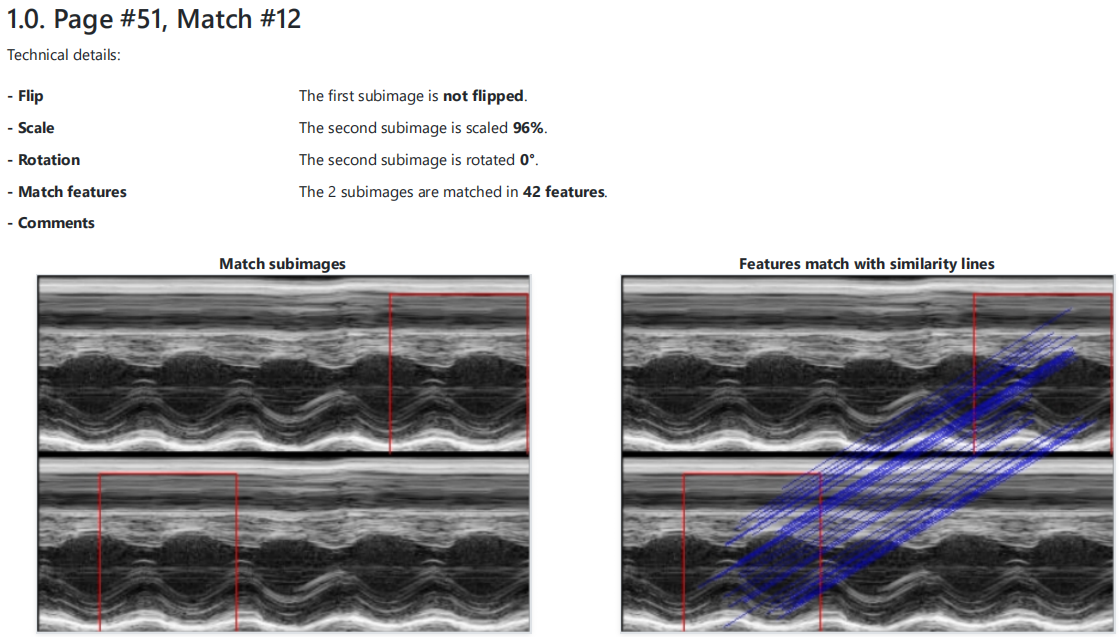


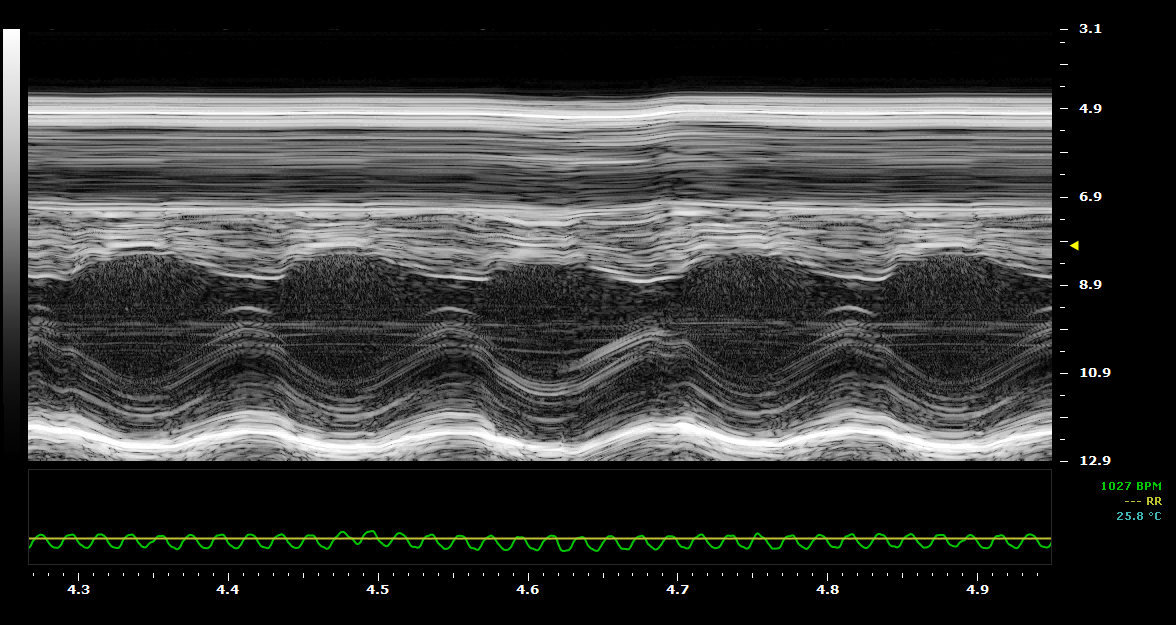


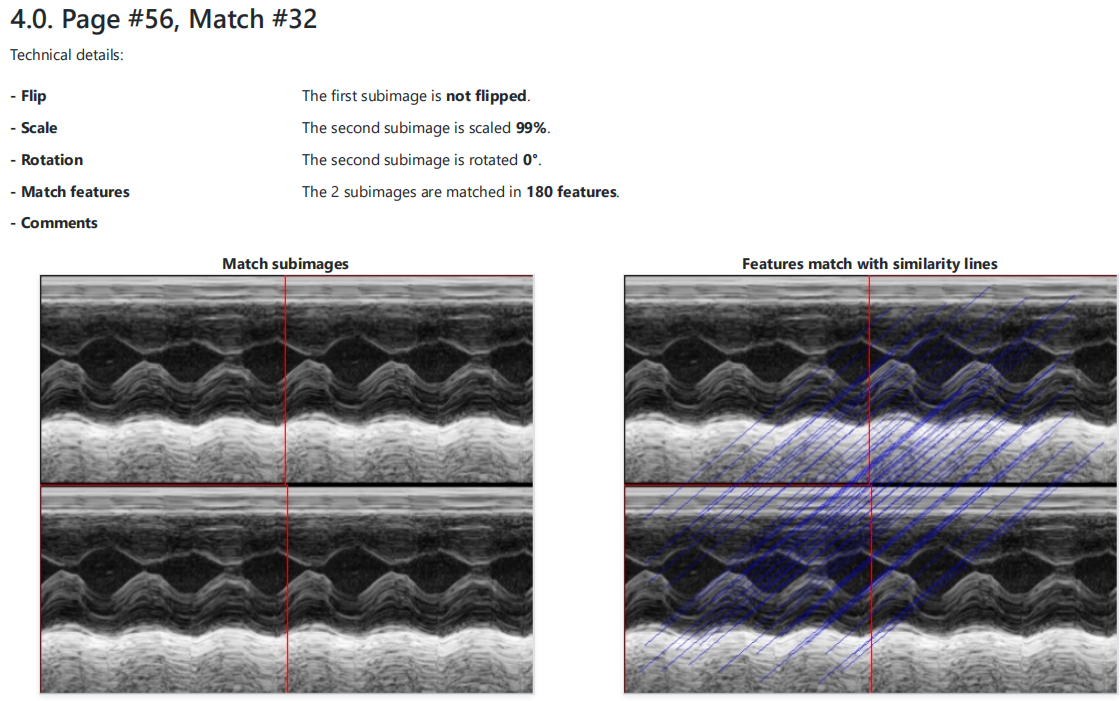


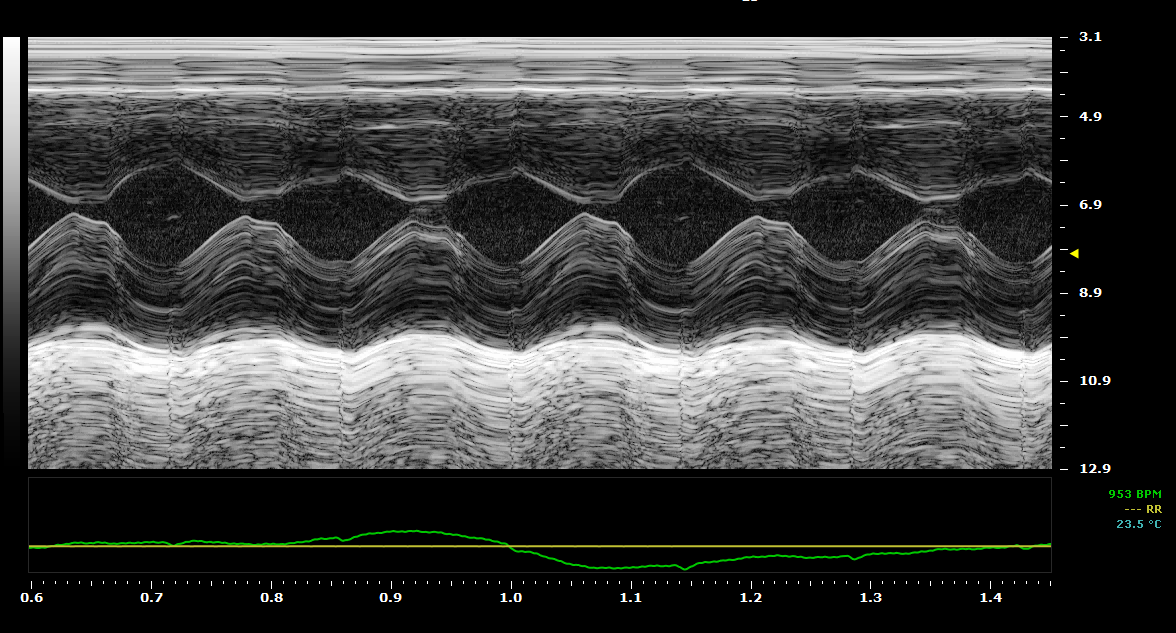


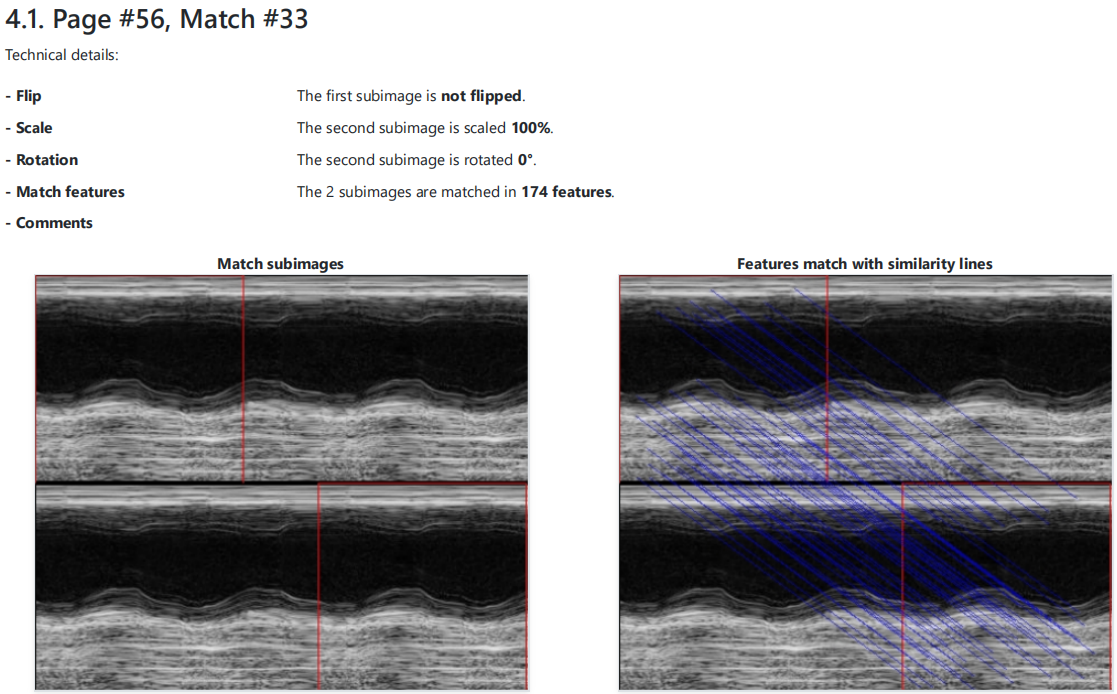


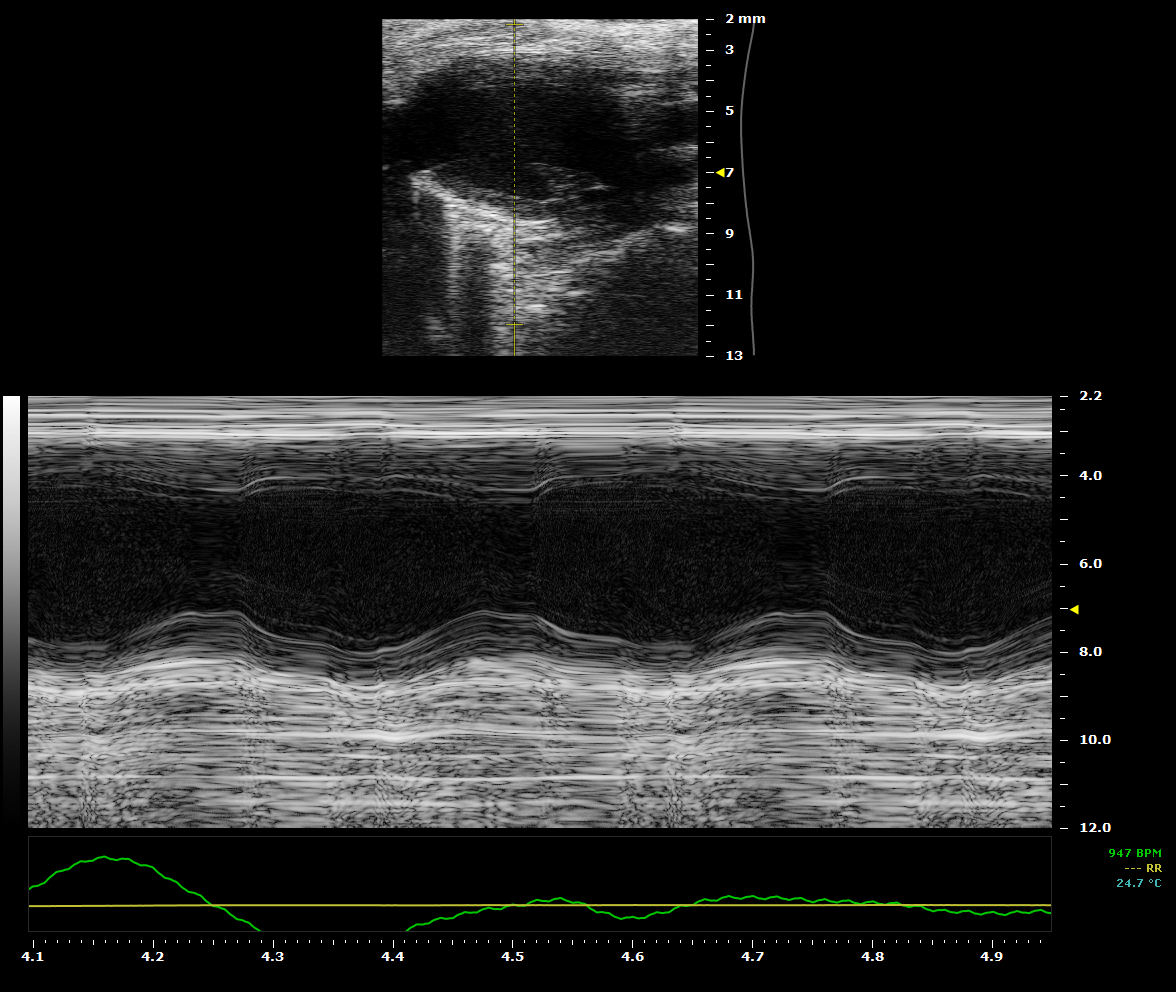


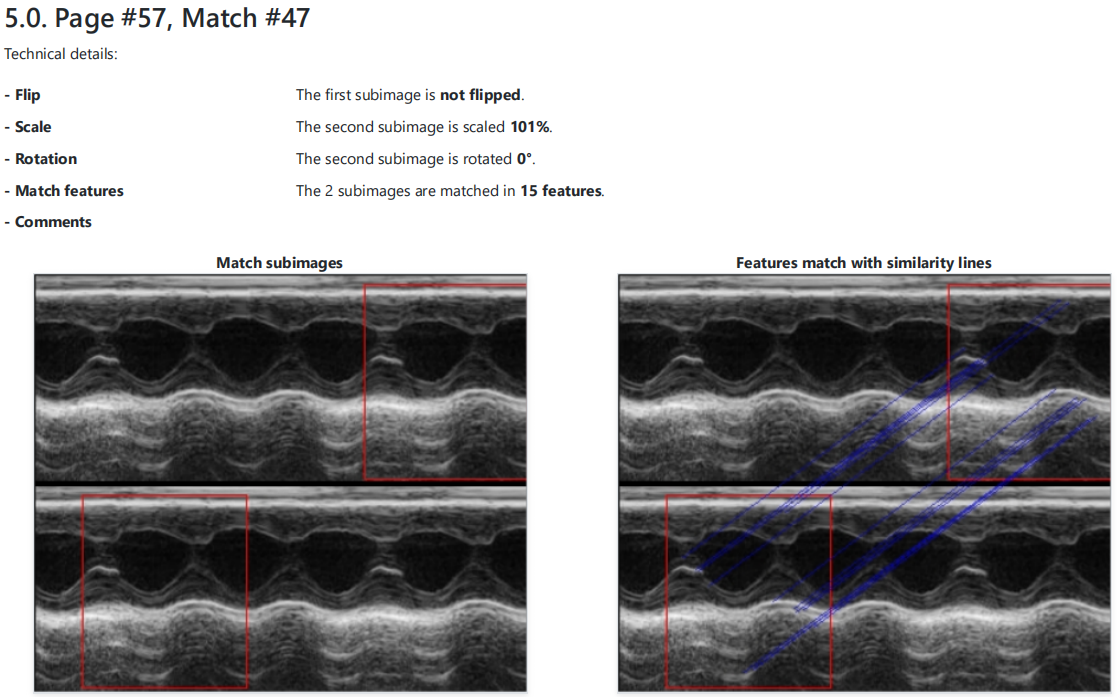

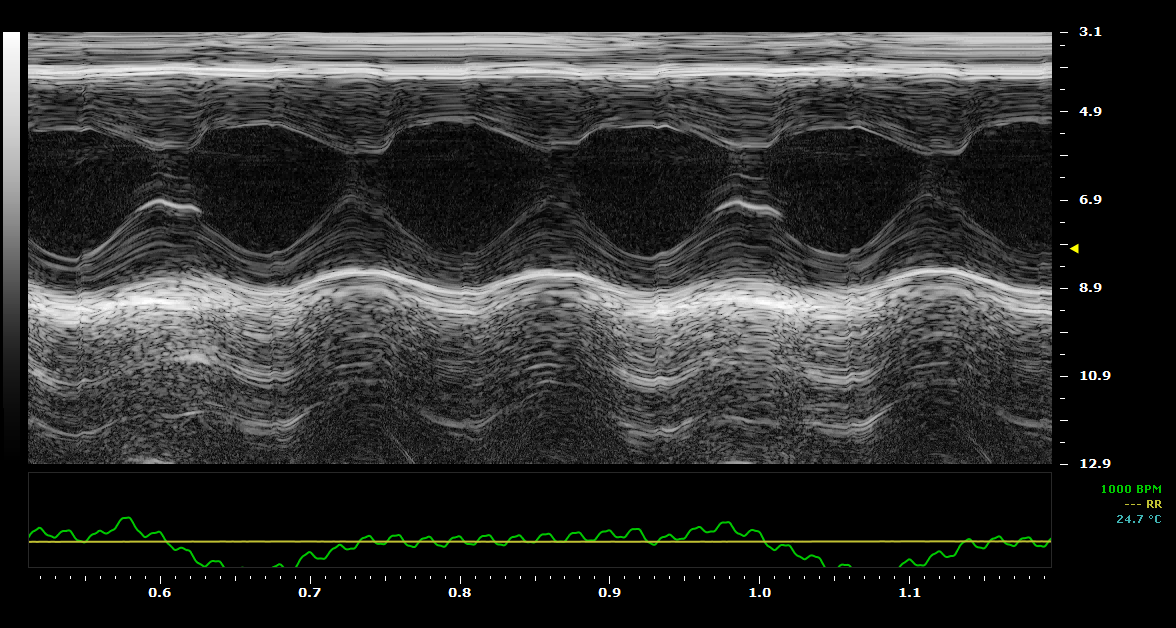


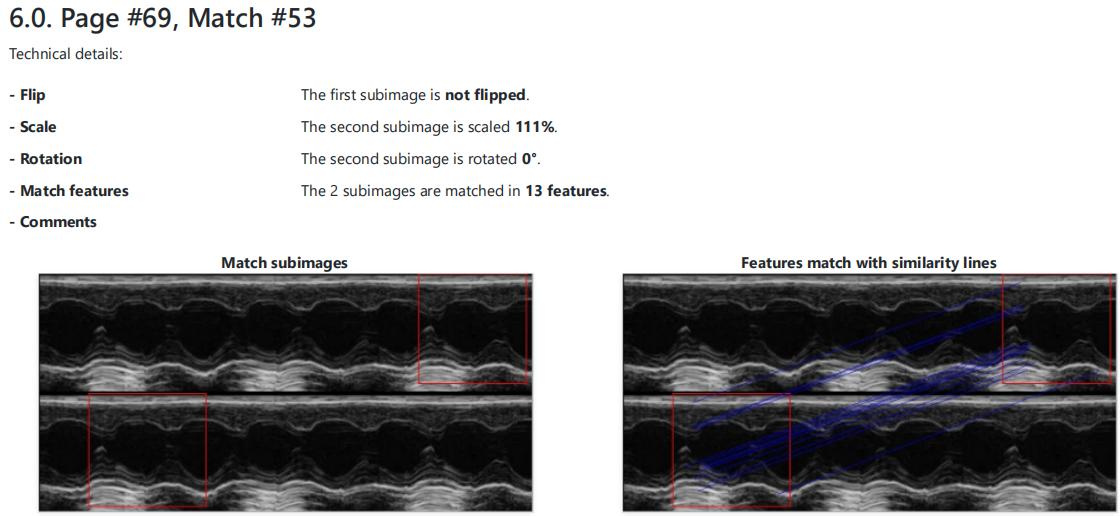


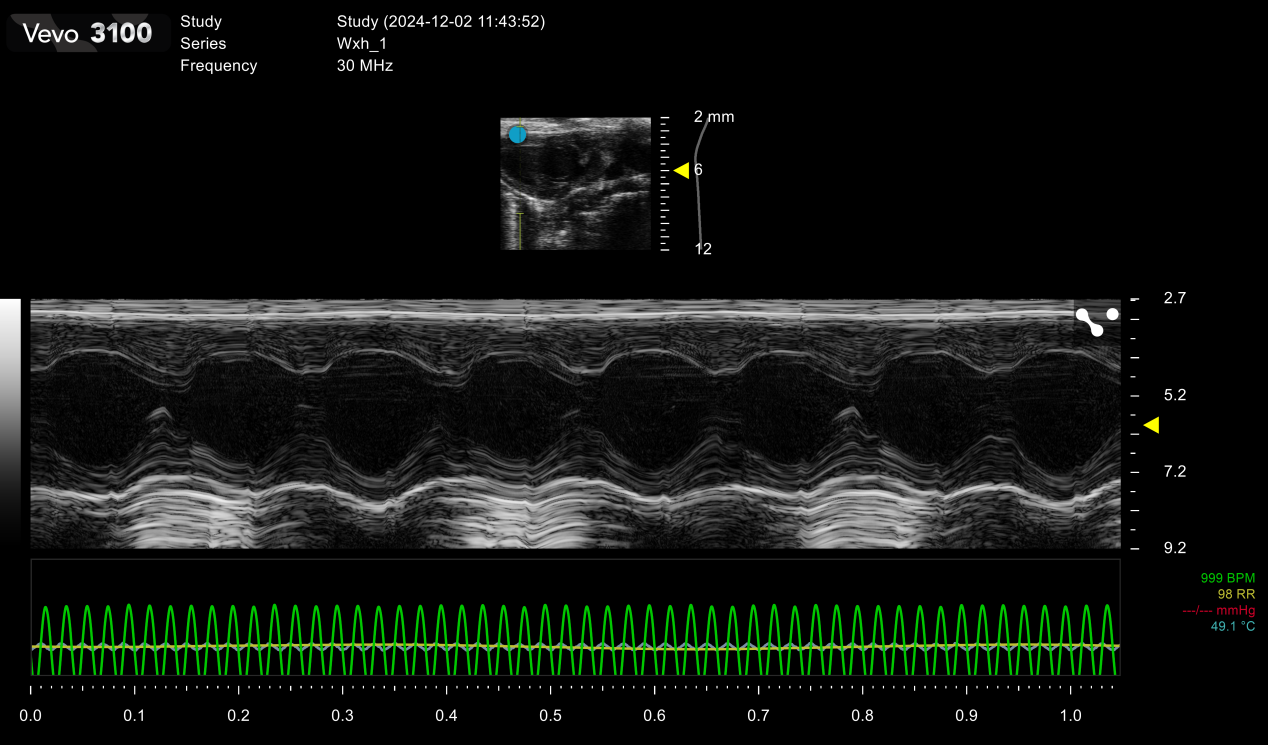

Supplement: Supplementary file 2 — RAW Doubtful Echocardiography [file 41392_2025_2216_MOESM2_ESM.docx]

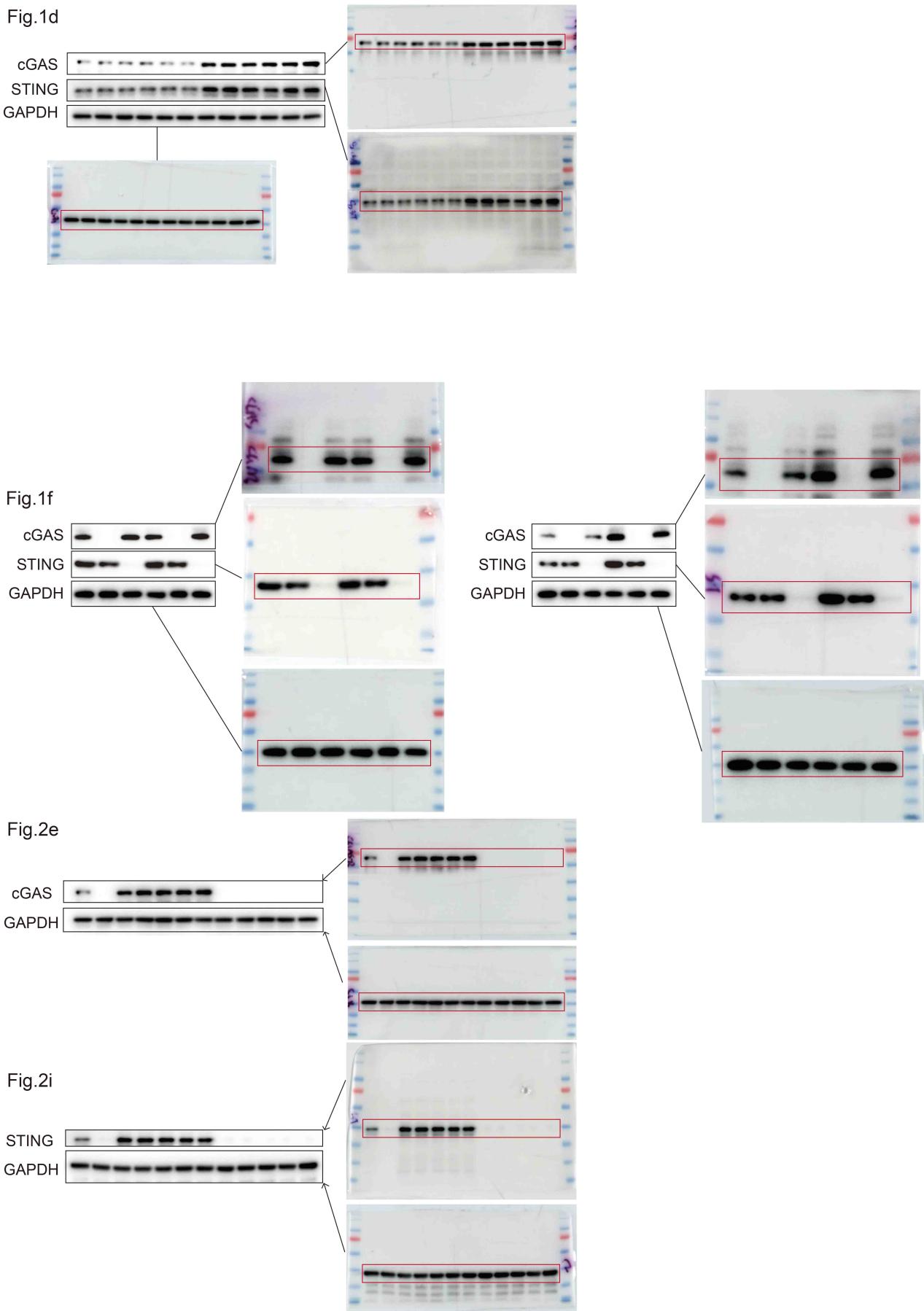

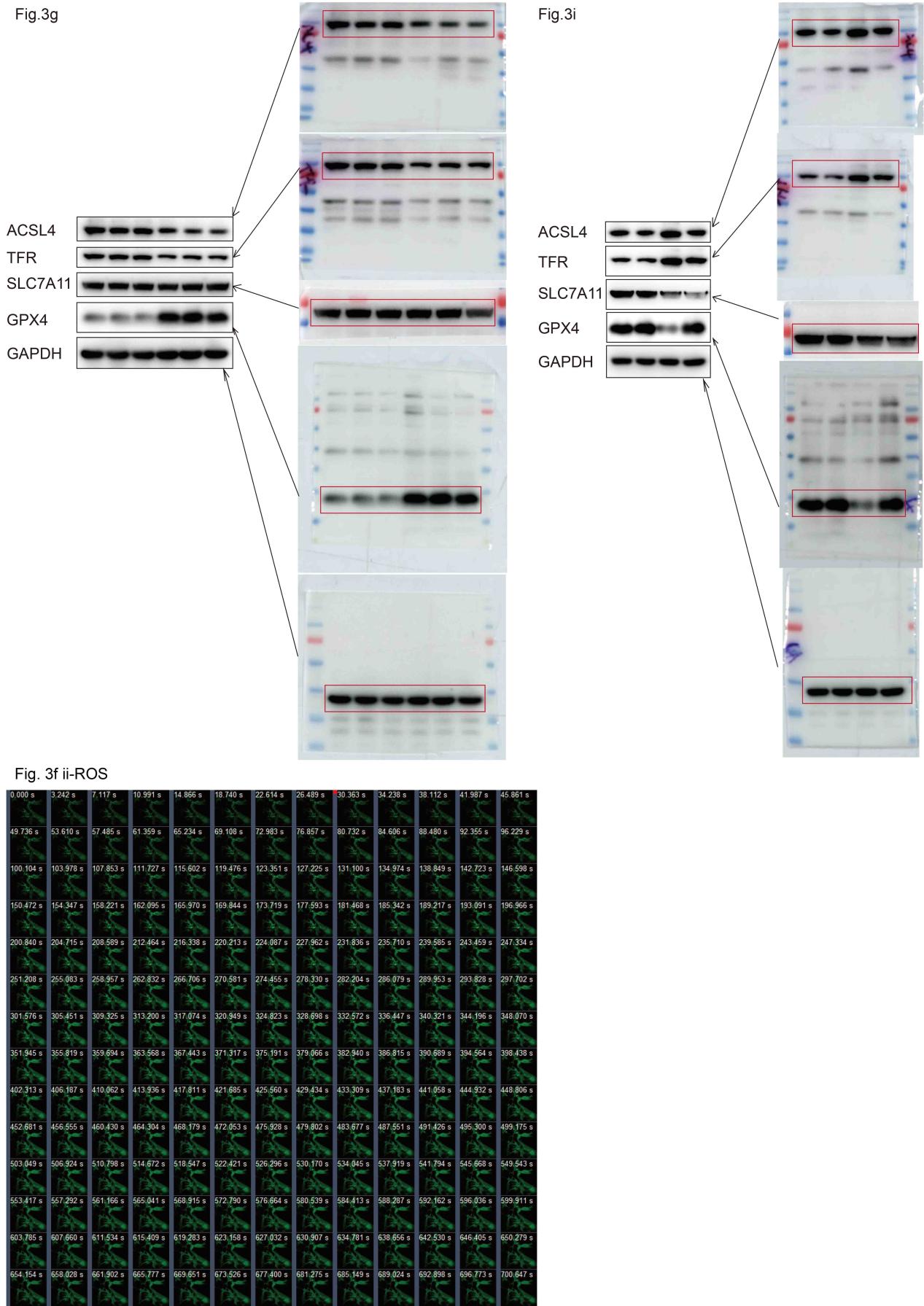

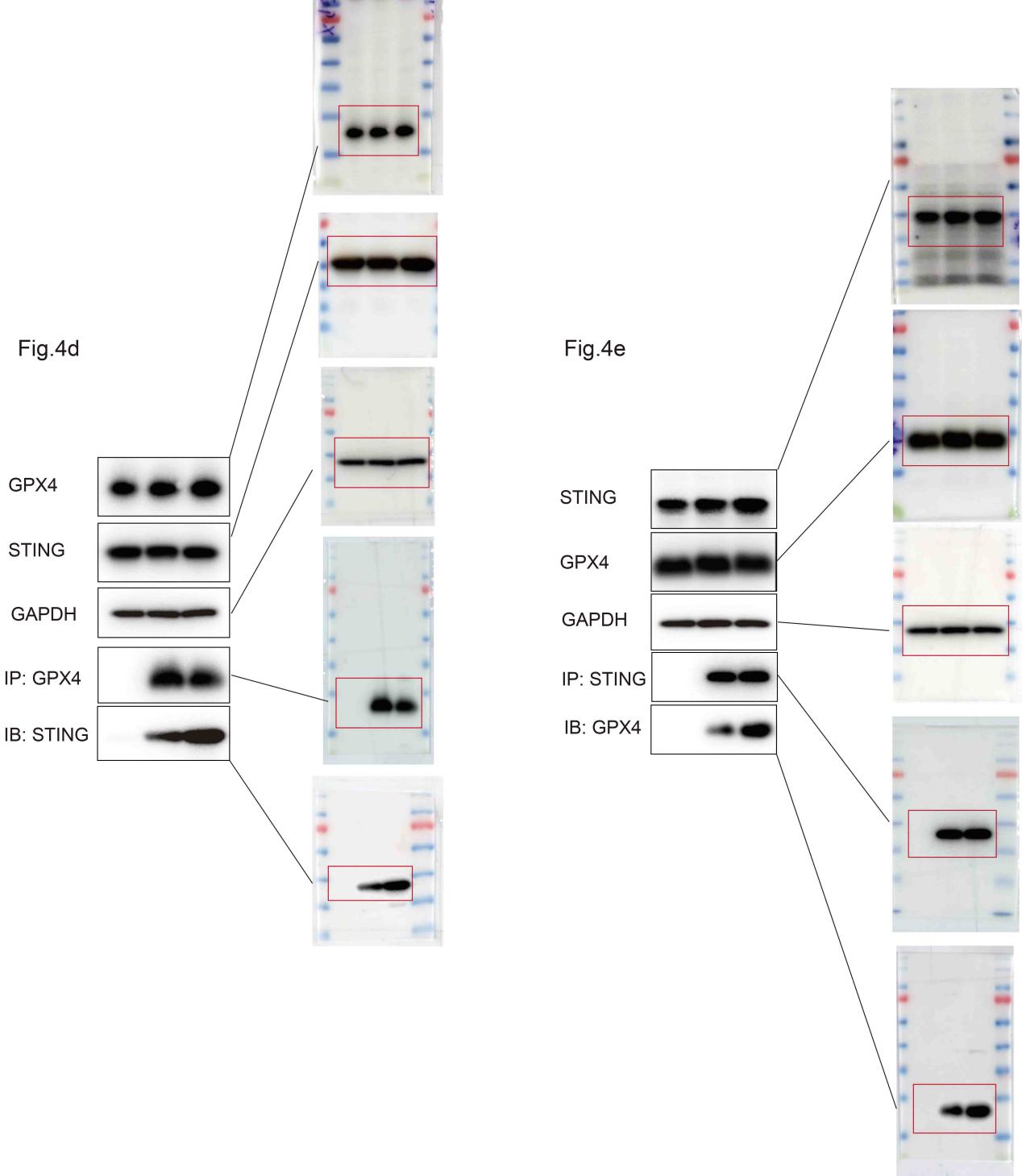

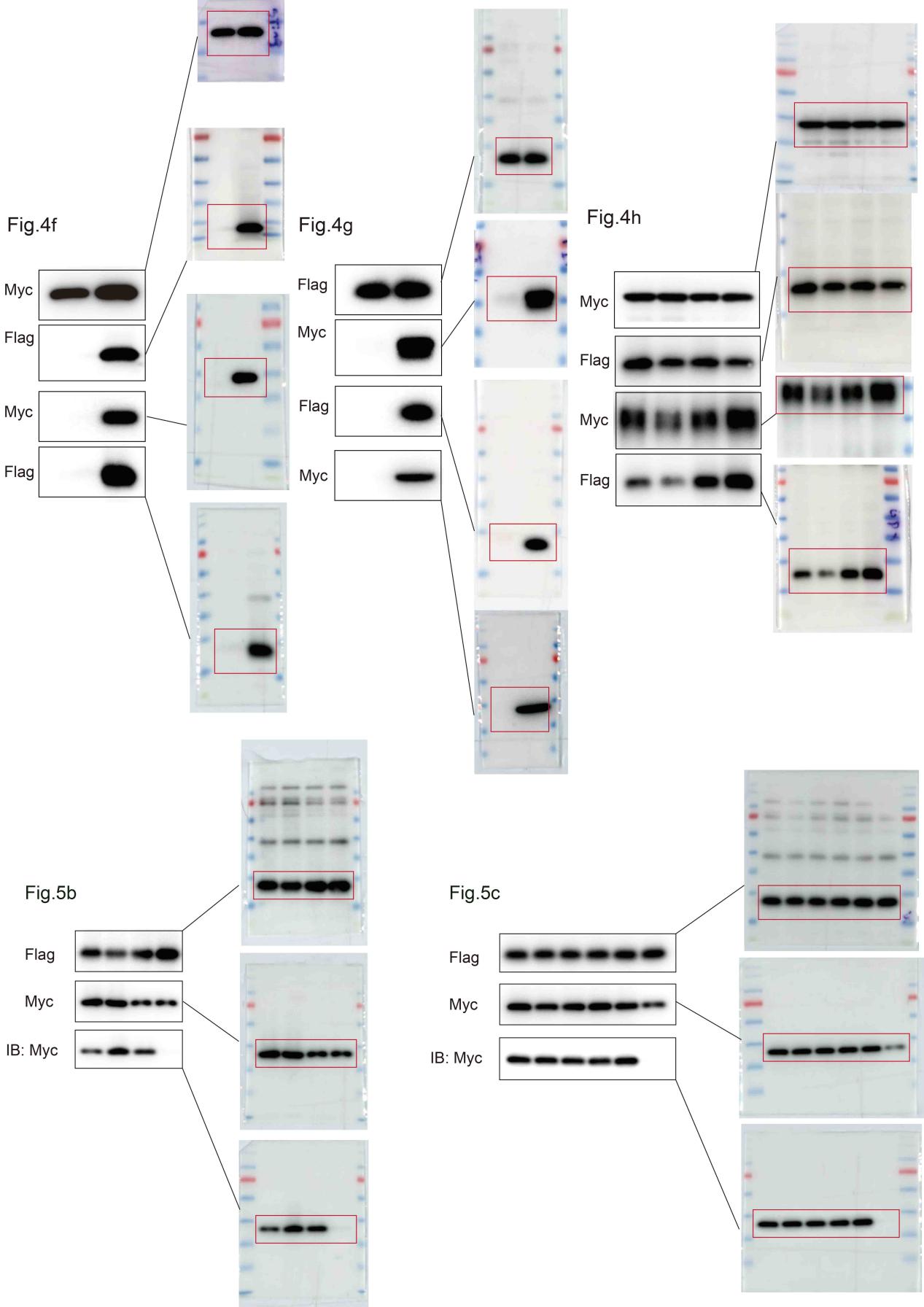

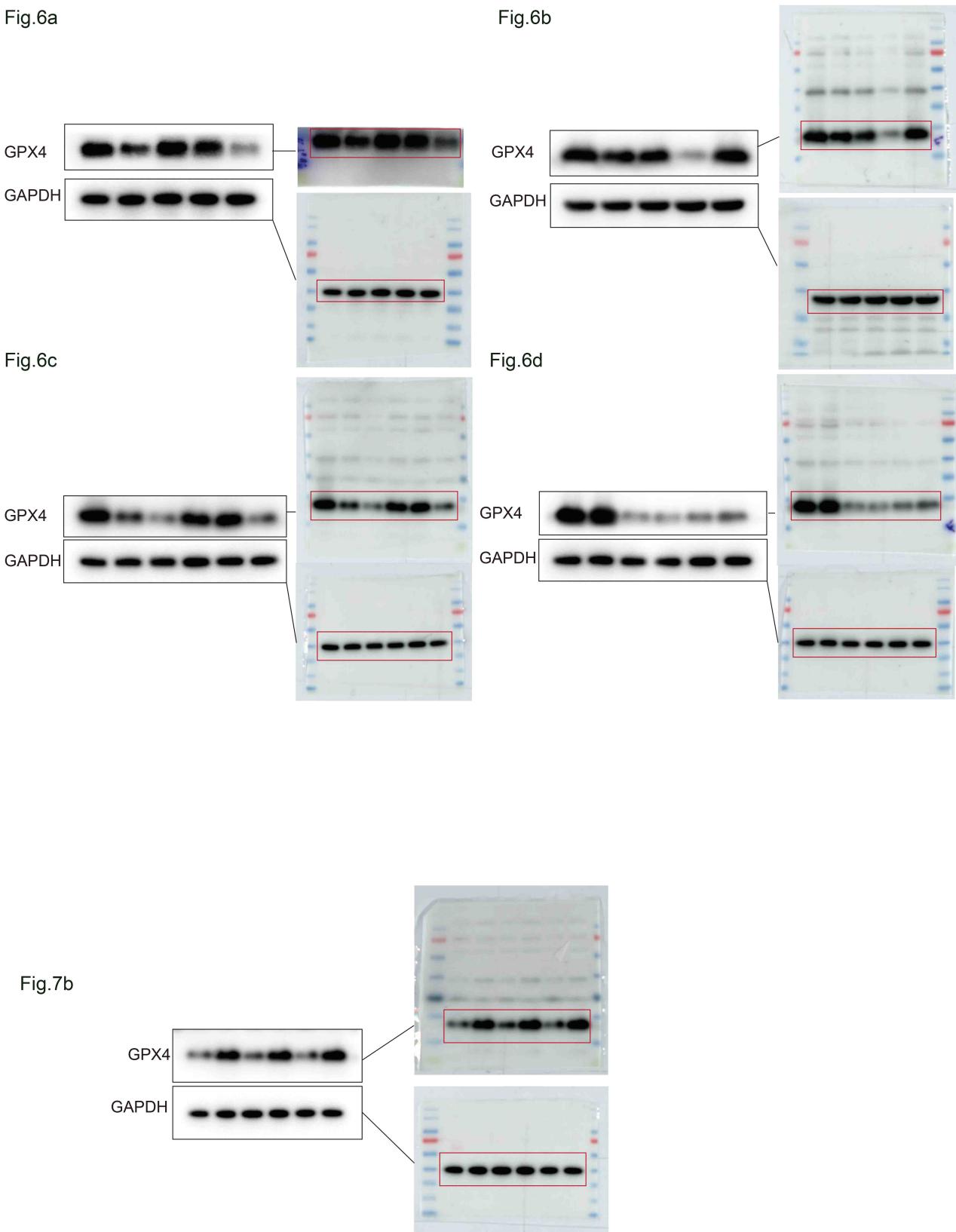

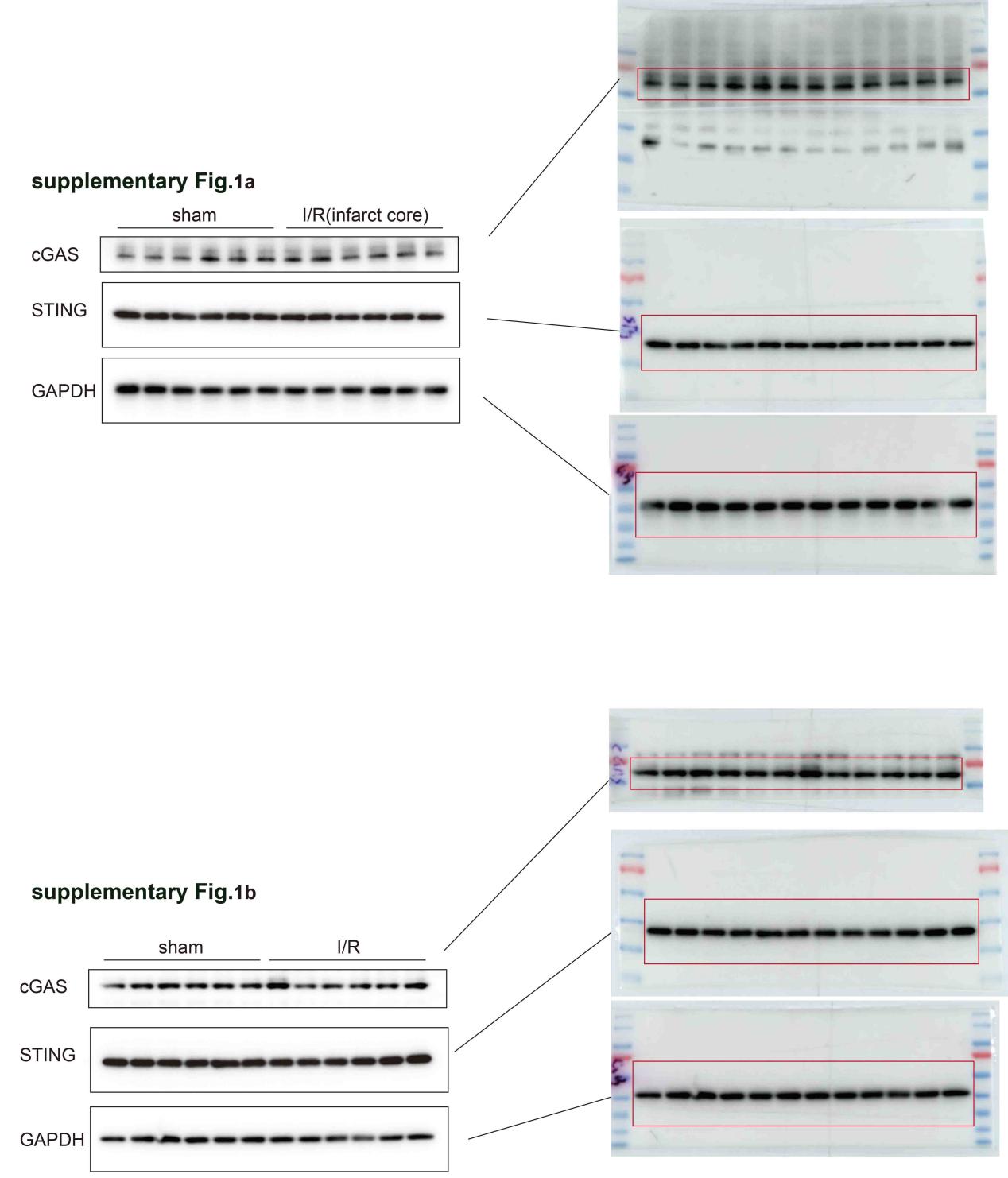

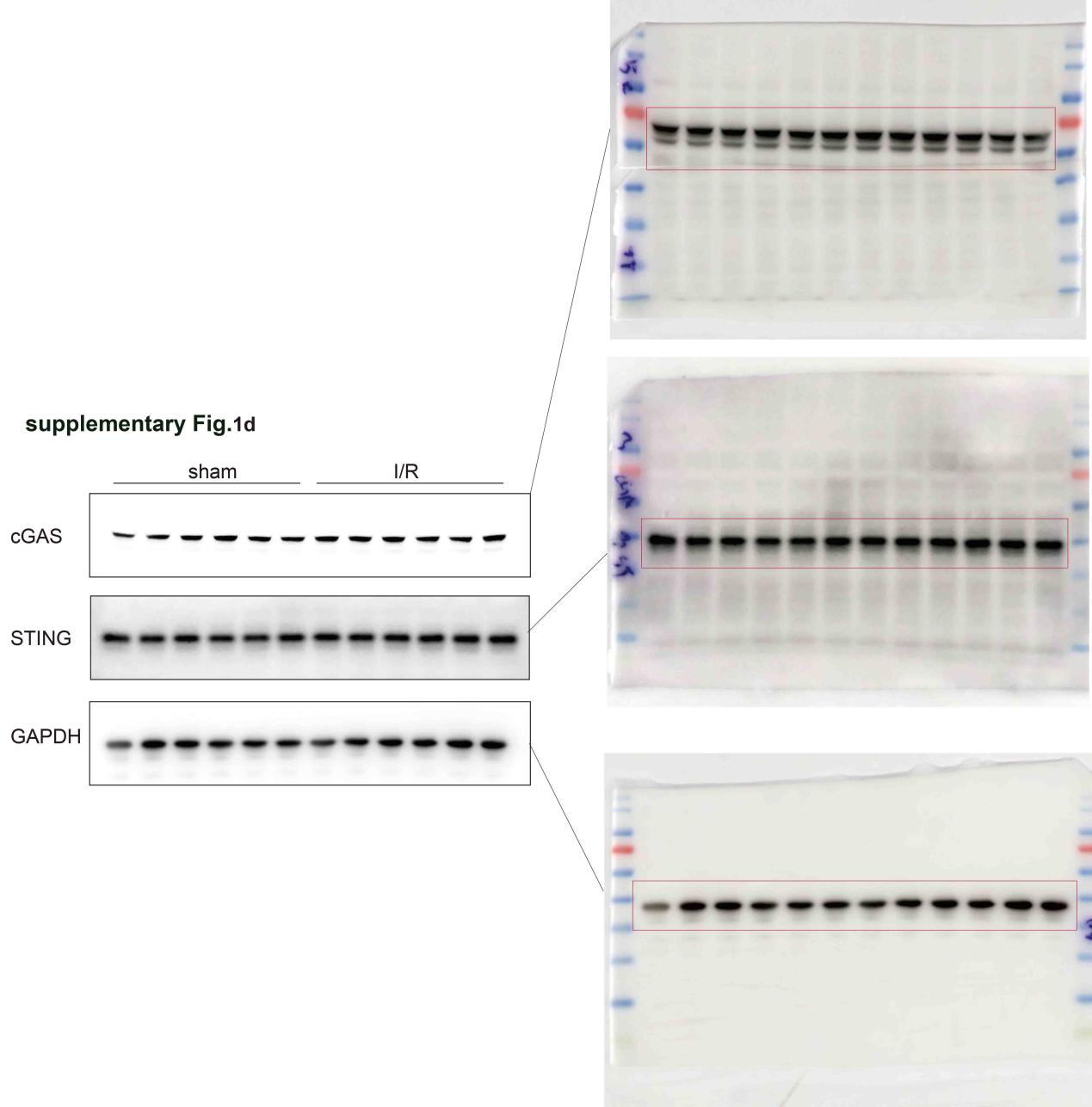

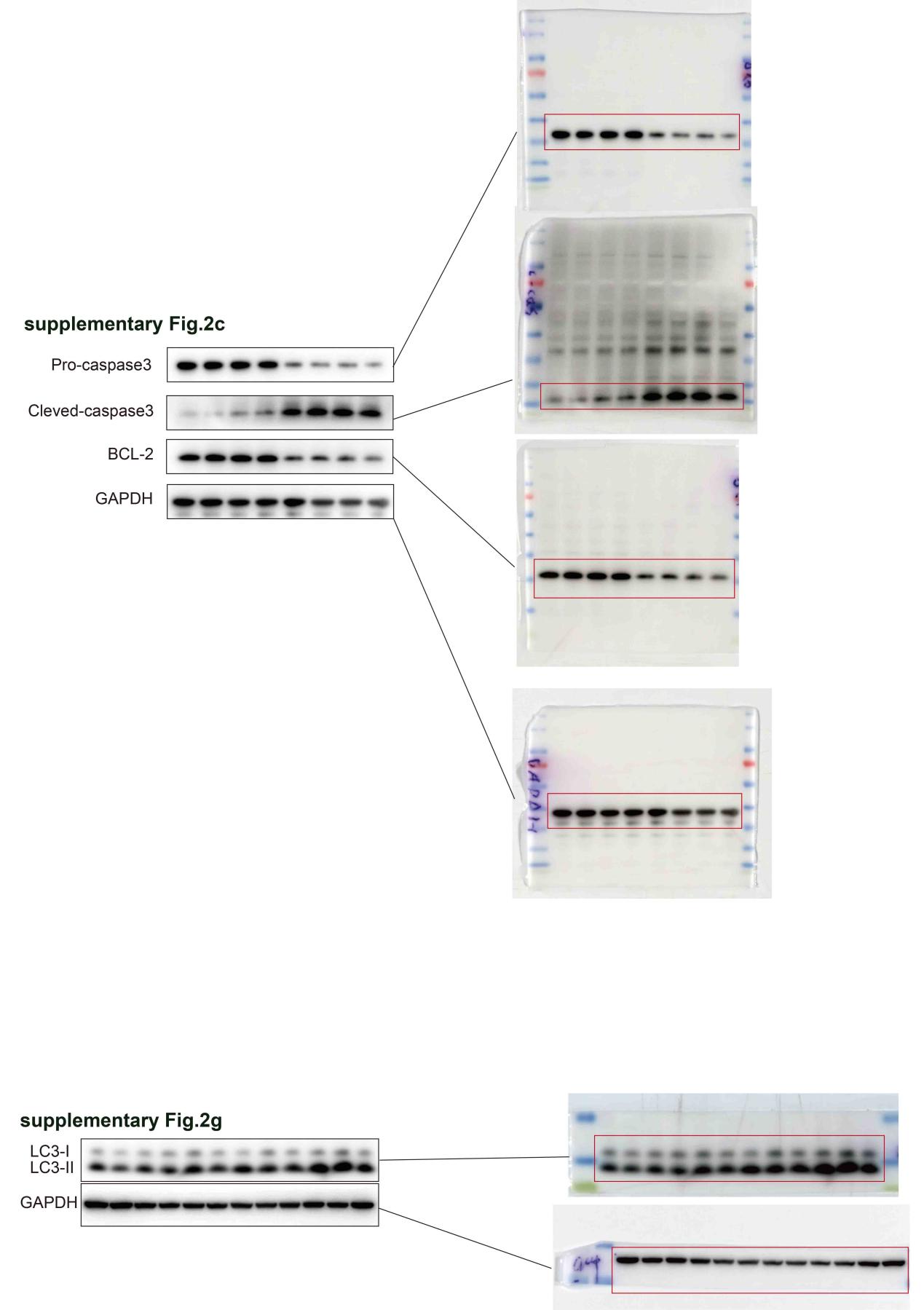

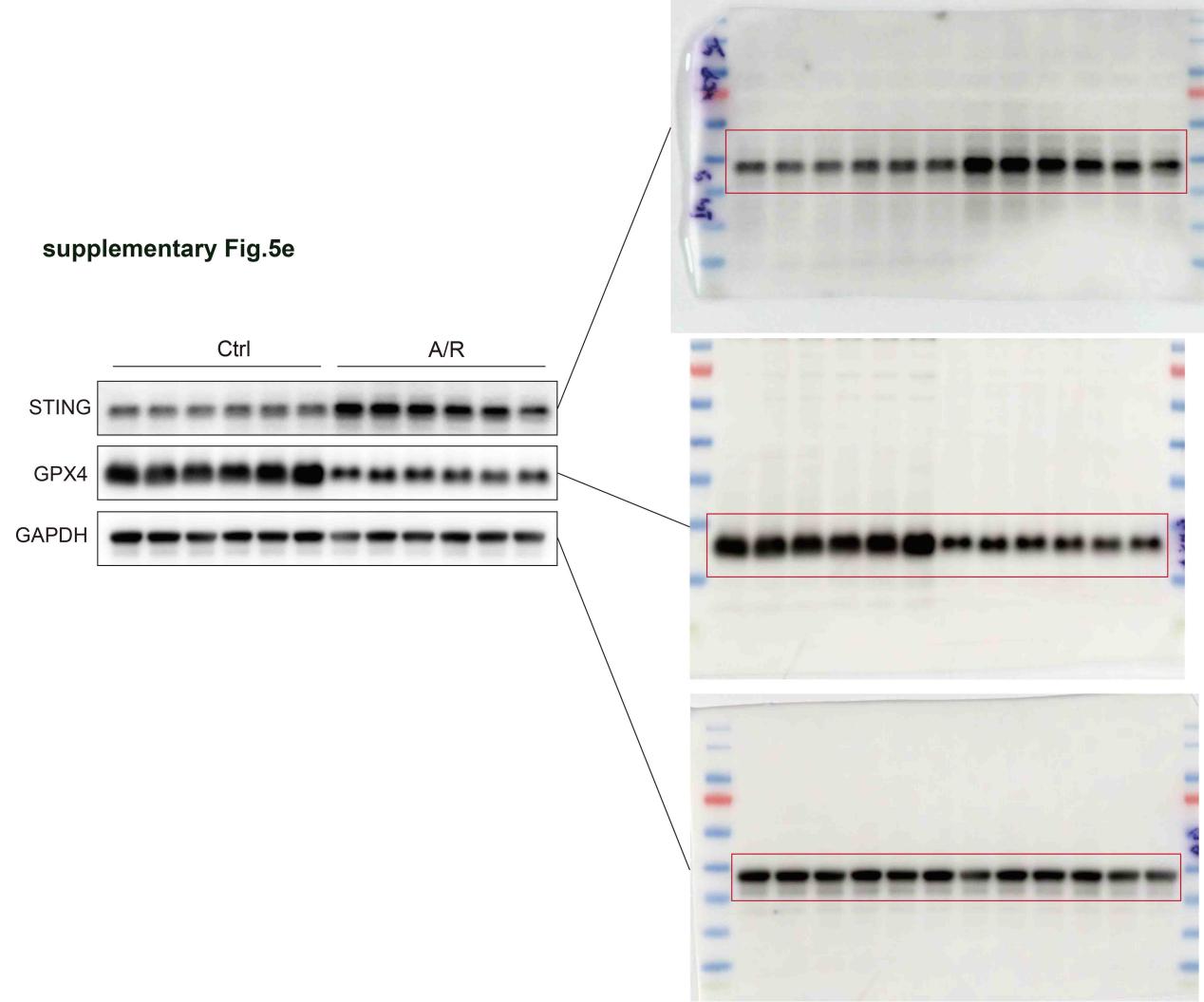

Supplement: Supplementary file 3 — RAW WB [file 41392_2025_2216_MOESM3_ESM.docx]
